# Addressing Oxygen Embrittlement in Additively Manufactured Titanium via Cu‐Mediated Interstitial Site Engineering

**DOI:** 10.1002/advs.202519184

**Published:** 2026-01-29

**Authors:** Xiaobin Lin, Xudong Rong, Jiachen Xie, Xinru Wang, Jianteng Wang, Zhihang Xu, Dongdong Zhao, Shiwei Pan, Feng Qian, Longlong Ma, Gang Sha, Xiang Zhang, Chunsheng Shi, Chunnian He, Naiqin Zhao

**Affiliations:** ^1^ Tianjin Key Laboratory of Composite and Functional Materials, School of Materials Science and Engineering Tianjin University Tianjin People's Republic of China; ^2^ State Key Laboratory of High Performance Roll Materials and Composite Forming Tianjin University Tianjin People's Republic of China; ^3^ State Key Laboratory of Precious Metal Functional Materials Tianjin University Tianjin People's Republic of China; ^4^ National Key laboratory of Science and Technology on Materials Under Shock and Impact, School of Materials Science and Engineering Beijing Institute of Technology Beijing People's Republic of China; ^5^ Herbert Gleiter Institute of Nanoscience, School of Materials Science and Engineering Nanjing University of Science and Technology Nanjing People's Republic of China

**Keywords:** additive manufacturing, dislocation behavior, hexahedral oxygen interstitial site, mechanical property, titanium alloy

## Abstract

The strength‐ductility trade‐off in oxygen‐containing titanium alloys has long been limited by the embrittling nature of octahedral interstitial oxygen (oct‐O). Herein, by integrating controlled laser powder bed fusion (L‐PBF) processing with Cu─O co‐alloying, we achieve, for the first time, the thermodynamic stabilization of hexahedral oxygen (hex‐O) configurations, which redefines the role of oxygen in titanium alloys. We showcase such interstitial engineering of oxygen relies on two key regimes: (1) Cu‐induced charge redistribution creates an electronic environment that preferentially stabilizes hex‐O sites through strong d‐p orbital hybridization, (2) rapid solidification process enabled by L‐PBF effectively suppresses the Ti─Cu excessive eutectoid reaction, preserving the integrity of strong Cu─O dipole chemical bonds. Mechanistically, hex‐O enhances < ***c*** >‐component dislocation activity through localized lattice distortion while maintains effective strain hardening via long‐range interactions with dislocations. This atomic‐scale manipulation in interstitial O enables an unprecedented strength‐ductility synergy of the titanium alloy, with a yield strength of 1121 MPa and a fracture elongation of 10.2%. Our work demonstrates a new pathway for tailoring the mechanical properties of oxygen‐tolerant titanium alloys via interstitial engineering.

## Graphical abstract

A novel “Cu‐mediated interstitial site engineering” strategy is introduced to thermodynamically stabilize hexagonal oxygen (hex‐O) configurations in oxygen‐containing titanium alloys. The hex‐O configuration activates multiple slip systems and enhances dislocation pinning via long‐range repulsion. This work offers a sustainable and cost‐effective pathway for fabricating high‐performance oxygen‐strengthened titanium alloys.

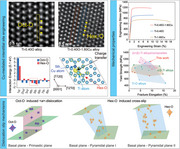

## 1 Introduction

The strategic incorporation of oxygen as an interstitial element in titanium alloys offers a cost‐effective pathway for achieving remarkable strength enhancement, as its solution strengthening efficiency surpass that of substitutional elements by orders of magnitude [1, 2, 3]. This strengthening mechanism originates from the oxygen‐induced asymmetric lattice distortions, which create localized stress fields that impede dislocation motion [4, 5, 6].

Despite the considerable efficacy as a solid‐solution strengthener, the introduction of oxygen into titanium alloys can cause embrittlement. Such detrimental effect has posed a major constraint on developing high‐oxygen‐content titanium alloys [7, 8, 9, 10], culminating in an inherent and challenging strength‐ductility trade‐off. The underlying physics of this limitation originates from the thermodynamic preference of oxygen atoms to occupy octahedral sites (oct‐O) within the α‐Ti matrix, where they accommodate an optimal interstitial volume and minimize strain energy, thereby stabilizing the Ti matrix [11]. While these oct‐O atoms effectively imped dislocation glide, they also induce persistent dislocation pinning through strong interactions with dislocation cores, leading to critical stress concentration [12, 13]. More profoundly, oct‐O can be shuffled by < ***a*** >‐type screw dislocations, which promotes planar slip behavior and exacerbates embrittlement [14, 15].

Altering the occupancy of octahedral interstitial oxygen atoms may offer an effective routine for mitigating oxygen embrittlement in titanium alloys, as modifications in site occupancy can modulate the local stress field, thereby influencing dislocation behavior and ultimately changing mechanical properties. Although limited investigations have investigated the potential for oct‐O to migrate to alternative interstitial sites in titanium, such as tetrahedral (tet‐O) and hexahedral (hex‐O) positions, to facilitate deformation coordination, their practical application is hindered by substantial energy barriers and inherent thermodynamic instability [16, 17]. Consequently, addressing the mechanical paradox of oxygen‐containing titanium alloys by regulating the interstitial positions of oxygen atoms not only holds as an open problem to explore but also remains a significant challenge to surmount.

Inspired by the above, we focused on leveraging the chemical interactions between copper and oxygen. Recent simulation efforts demonstrate that substitutional solute elements can modulate oxygen interstitial site occupancy through mutual chemical interactions. Notably, copper atom exhibits the strongest repulsion with oct‐O in α‐Ti environments (0.57 eV/atom) [18], outperforming other alloying candidates [17, 18, 19, 20]. This feature makes copper the promising candidate for destabilizing oct‐O configurations and facilitating interstitial site transitions. Nevertheless, conventional equilibrium processing methods, e.g. casting, for fabricating titanium alloys face significant limitations in effectively utilizing copper for altering the site occupancy of oct‐O. The slow cooling of these methods typically leads to spontaneous copper segregation into the β‐Ti phase or forming Ti‐Cu intermetallics [21, 22], thereby diminishing the chemical interaction between copper and oxygen atoms. Encouragingly, additive manufacturing (AM) emerges as a transformative solution to this problem due to its high cooling rate (10^3^–10^8^ K/s) and dynamic solidification conditions [23, 24, 25, 26, 27]. The ultrafast solidification achieved through AM is anticipated to suppress copper segregation and partially retain copper atoms within the α‐Ti phase via non‐equilibrium solidification. The anticipated copper‐oxygen chemical interaction is expected to manipulate the interstitial oxygen position, hence the mechanical properties of Ti alloys.

Leveraging these advantages, we successfully employed selective laser melting (SLM) to fabricate Ti‐0.40 wt.% O‐1.60 wt.% Cu alloy (Ti‐0.40O‐1.60Cu) with controlled oxygen interstitial engineering. Our findings reveal that Cu─O coordination, mediated by adjacent titanium atom, stabilizes hex‐O sites as a previously unreported configuration in oxygen‐containing titanium alloy. During deformation, this unique hex‐O site not only remains positionally stable under external stress but also exerts substantial repulsive forces on dislocation cores. Such strong repulsion significantly enhances the pinning effect on dislocations, directly elevating the alloy's strength without sacrificing structural integrity. Particularly, the repulsion between hex‐O and dislocation can effectively activate multiple < ***c*** + ***a*** > slip systems that are typically inert in conventional Ti─O alloys. It is this activation that further induces the nucleation and propagation of < ***c*** >‐component dislocation, which enable cross‐slip across different slip planes and accommodate more plastic strain. The dual effects directly translate to a remarkable optimization of mechanical properties of the SLM‐fabricated Ti‐0.40O‐1.60Cu alloy, wherein a simultaneous increase in strength and uniform elongation compared to both traditional oxygen‐strengthened titanium alloys and SLM‐fabricated pure Ti was achieved. This breakthrough establishes a novel paradigm for atomic‐scale manipulation of interstitial oxygen sites, offering a promising strategy to address the long‐standing challenge of oxygen embrittlement in oxygen‐strengthened titanium alloys.

## 2 Results and Discussion

### 2.1 Formation Mechanism of the Cu‐Mediated Hexahedral Interstitial Oxygen

**FIGURE 1:**
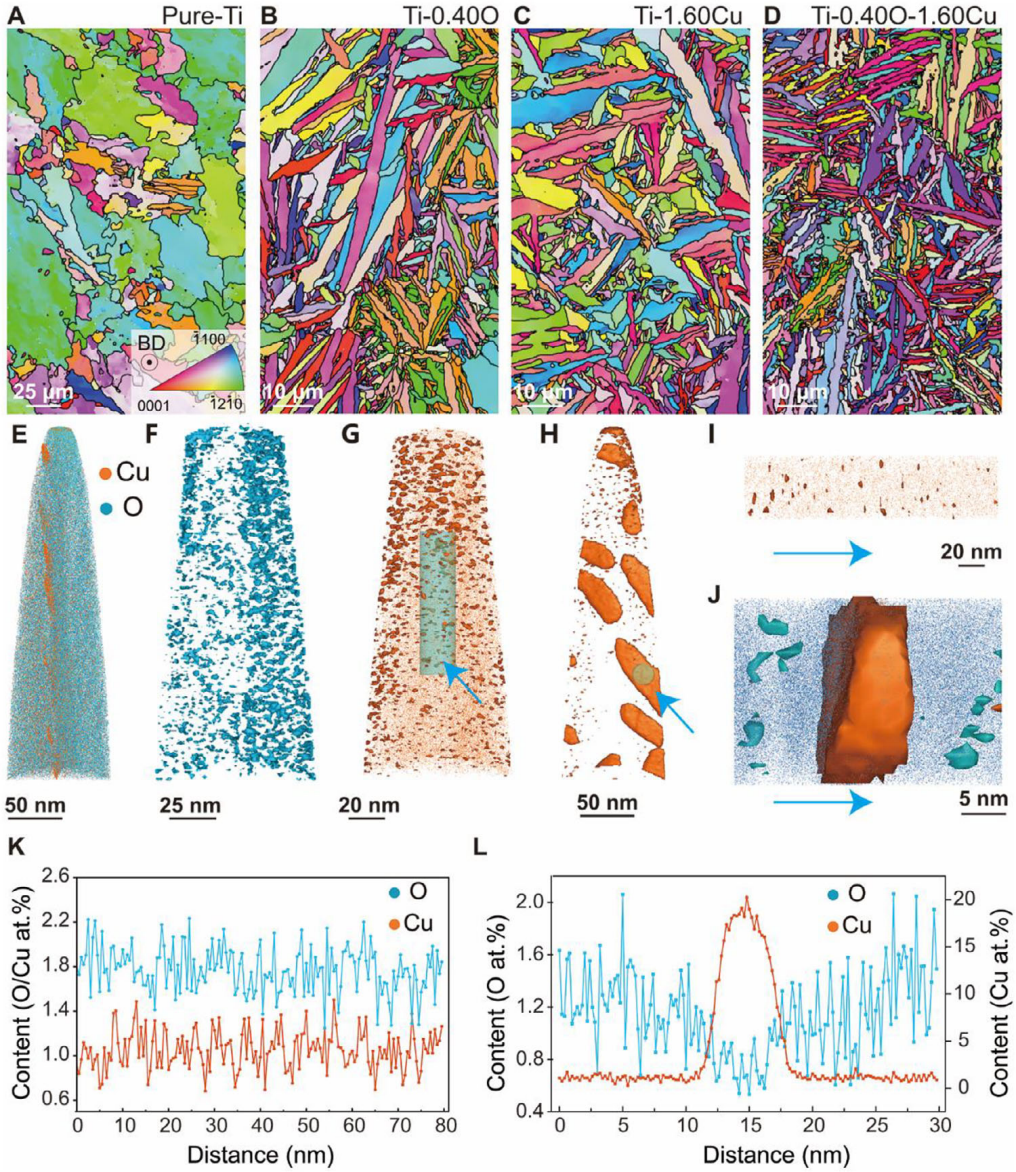
Microstructure characterizations. The IPF maps of the (A) Pure‐Ti, (B) Ti‐0.40O alloy, (C) Ti‐1.60Cu alloy and (D) Ti‐0.40O‐1.60Cu alloy, respectively, oriented perpendicular to the building direction (BD). (E)∼(I) APT characterizations of the Ti‐0.40O‐1.60Cu alloy. (E) 3D atomic reconstruction maps. (F) O atom distribution diagram for the 3.0 at.% iso‐composition surface in α/α′‐lath. (G) Cu atom distribution diagram for the 2.5 at.% iso‐composition surface associated with (F). (H) Cu atom distribution diagram for the 3.5 at.% iso‐composition surface associated with (E) from another perspective. (I) The partial enlarged view of the marked area in (G). (J) The partial enlarged view of the marked area in (H). (K) The content statistics of O and Cu elements along the blue arrow direction in (I). (L) The content statistics of O and Cu elements along the blue arrow direction in (H).

The inverse pole figure (IPF) maps determined by Electron backscatter diffraction (EBSD) reveal the grain microstructures of the pure‐Ti, Ti‐0.4O, Ti‐1.60Cu, Ti‐0.40O‐1.60Cu alloy (Figure 1A–D) oriented perpendicular to the building direction (BD). The microstructure of pure‐Ti consists of relatively coarse α/α′ grains, with an average grain size of 6.48 µm (Figure 1A; Table S1). The addition of 0.40 wt.% O or 1.60 wt.% Cu to pure‐Ti results in a significant alteration of the grain microstructure. Numerous α/α′‐lath structures are observed in the Ti‐0.40O and Ti‐1.60Cu alloys, indicated by the average width of 1.03 and 1.21 µm, respectively (Figure 1B,C; Table S1). Notably, the Cu─O co‐alloying gives rise to the formation of refined lamellar α/α′‐lath structures in the Ti‐0.40O‐1.60Cu alloy characterized by an average width of 0.90 µm and length of 2.78 µm (Figure 1D). These observations indicate the synergistic effects of Cu and O elements in retarding grain growth. The α/α′‐lath structures in the Ti‐0.40O‐1.60Cu alloy were further observed using transmission electron microscopy (TEM) in Figure S1A. The energy‐dispersive spectroscopy (EDS) map in Figure S1B clearly reveals the enrichment of Cu atom at theα/α′‐lath interface. Atom probe tomography (APT) analysis of the region marked in Figure S1A further elucidates the atomic‐scale distribution of O and Cu elements. The 3D reconstruction of the Ti‐0.40O–1.60Cu alloy (Figure 1E) revealed a clear enrichment of O within the α/α′‐laths, whereas Cu was predominantly segregated at the α/α′‐lath rims. Since O atoms are intrinsic components of the α‐Ti phase, they tend to distribute more uniformly within the α/α′‐laths, as illustrated by the 3.0 at.% iso‐composition surface in Figure 1F. In addition, Cu atoms were also randomly distributed throughout the α/α′‐lath, with no obvious clustering or compositional segregation observed (Figure 1G). Figure 1I displays the partial enlarged view of the marked area in Figure 1G. The corresponding local line concentration analysis further confirms the uniform dispersion of O and Cu atoms (Figure 1K). By contrast, the 3.5 at.% Cu iso‐composition surface clearly visualized a higher concentration of Cu specifically at the α/α′ interface (Figure 1H). An enlarged view (Figure 1J) of the region highlighted by blue arrows in Figure 1H, together with the corresponding 1D concentration profiles in Figure 1L, clearly demonstrates that the enrichment of Cu atoms at the α/α′‐lath rims was accompanied by a depletion of O element.

**FIGURE 2:**
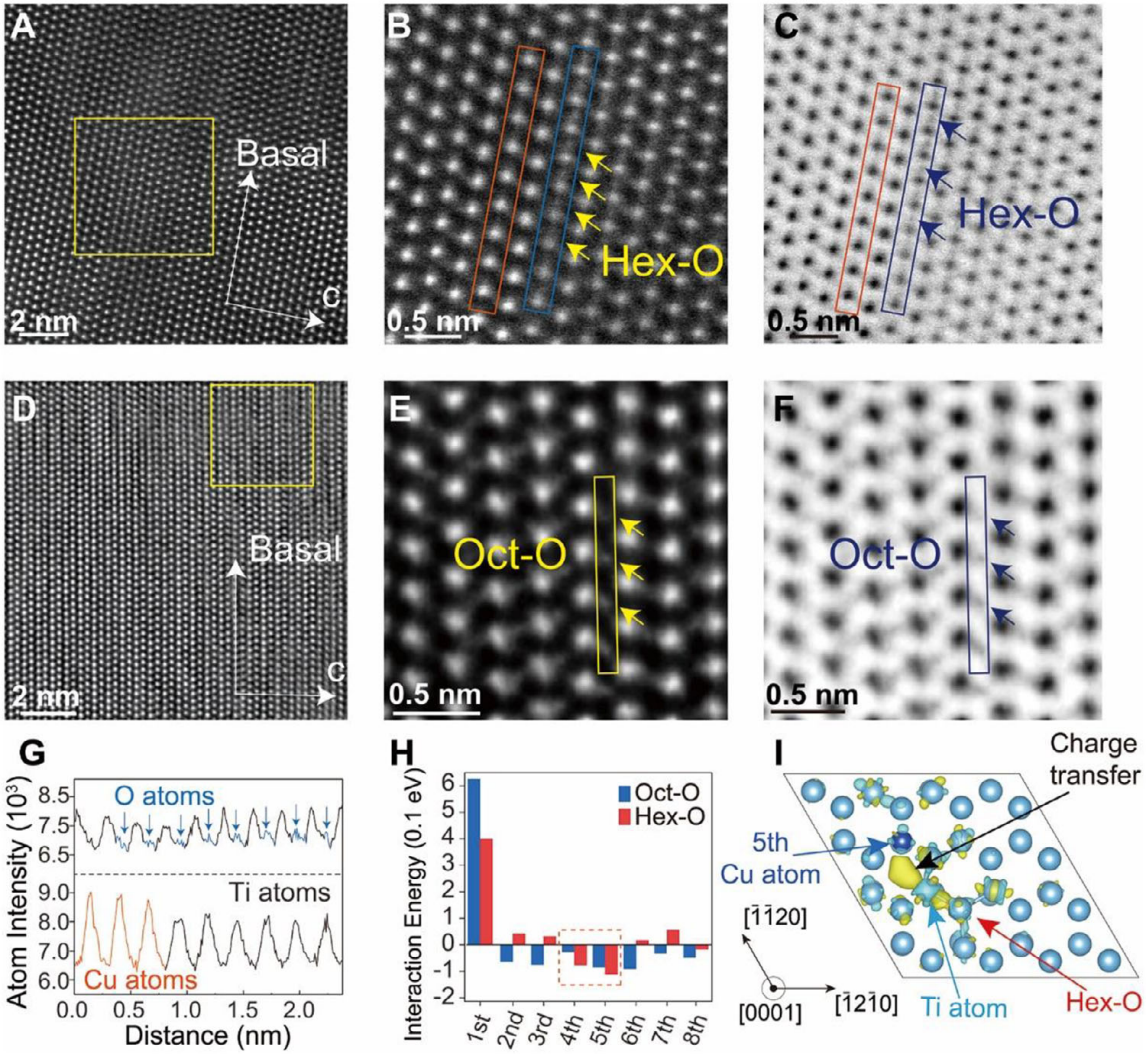
The characterizations of interstitial oxygen atoms. (A,B) The double aberration‐corrected STEM‐HAADF micrographs of Ti‐0.40O‐1.60Cu alloy along the [$11\bar{2}0$] zone axis showing the hex‐O distribution in the α/α′‐lath. (C) The bright‐field (BF) image of the Ti‐0.40O‐1.60Cu alloy according to (B). (D,E) The double aberration‐corrected STEM‐HAADF micrographs of Ti‐0.40O alloy along the [$11\bar{2}0$] zone axis showing the oct‐O distribution in the α/α′‐lath. (F) BF image of the Ti‐0.40O alloy according to (E). (G) The atomic intensity in orange and blue line profiles corresponding to the squared regions in (B), respectively. (H) The DFT calculation results of the interaction energy between O and Cu solutes at different positions with unequal distance. (I) The interfacial difference charge density of Ti‐0.40O‐1.60Cu alloy at iso‐surface level of 1.50 × 10^−4^ e/Bohr [3].

Double aberration‐corrected high‐angle annular dark‐field (HAADF) image obtained via scanning transmission electron microscopy (STEM), performed along the [$11\bar{2}0$] zone axis, directly resolves the atomic configuration within the α‐laths of the Ti‐0.40O–1.60Cu alloy (Figure 2A). Enlarged views (Figure 2B,C) and a corresponding line profile (Figure 2G) further demonstrate that O atoms occupy hexahedral interstitial sites located on the same basal plane with adjacent Ti atoms. The corresponding atomic model along the [$11\bar{2}0$] zone axis is presented in Figure S2A. Specifically, the constructed crystal model (Figure S2B) demonstrates that when O atoms are situated at the hexahedral interstitial sites, their atomic distribution in the {$11\bar{2}0$} plane is consistent with the characterization results shown in Figure 2B, which defines the distinctive microstructure of hex‐O. Critically, the line profile (Figure 2G) according to Figure 2B also exhibits signal intensity peaks substantially higher than those associated with Ti atoms. The Z‐contrast variation observed in the HAADF image corresponding to Figure 2B identifies these anomalous signals as originating from the positions of Cu atoms (Figure S2C). Besides, the enlarged view (Figure S2D) from APT analysis of the α/α′‐lath in the sample shown in Figure 1F further indicates that Cu atoms are predominantly distributed around the O atoms. In contrast, comparative analysis of the Ti‐0.40O alloy suggests conventional occupancy of O atoms at octahedral interstitial sites (Figure 2D–F). These atomic‐scale observations provide direct evidence supporting the Cu‐induced stabilization of the hex‐O configurations.

To elucidate the thermodynamic origin of hex‐O stabilization, the ultrafast cooling rate characteristic of L‐PBF, typically in the range of 10^3^ to 10^8^ K/s, exerts critical kinetic constraints that favor the trapping of Cu elements within the α/α′‐lath. In contrast to conventional casting processes, which involve slow cooling and prolonged diffusion times [28], the rapid solidification in L‐PBF effectively suppresses the phase separation of Cu and the formation of Ti‐Cu intermetallics. This is attributed to the limited time available for solute redistribution and the high undercooling‐induced nucleation of α/α′. As a result, a fraction of Cu atoms tends to segregate toward the α/α′ interfaces, forming localized Cu‐enriched regions. This Cu segregation creates a barrier to the migration of O atoms to the interfaces, enabling O atoms to be fully dissolved into the α/α′ interior. Moreover, the rest Cu atoms in the α/α′ interior modify the local energy environment for O atoms.The interaction behavior between Cu and O atoms in Ti matrix were probed using density functional theory (DFT) simulations [29]. Analysis of the atomic structure revealed that oct‐O possesses nine neighboring Ti positions, while hex‐O exhibits twelve within the same unit cell. The nine nearest neighbor positions of oct‐O and hex‐O are taken into consideration. These neighboring Ti atoms were systematically substituted with Cu atoms. The calculated interaction energies, summarized in Figure 2H and Table S2, demonstrate a preferential formation of hex‐O when coordinated with Cu at the fourth/fifth nearest‐neighbor positions, as indicated by the lowest interaction energy. Charge density difference analysis (Figure 2I; Figure S3A) reveals pronounced electron accumulation around hex‐O and Cu atoms, connected through a continuous Ti‐mediated charge bridge. The partial density of states (PDOS) (Figure S3B) demonstrates strong hybridization between Cu 3d and Ti orbitals (in the range of −4.0 to 1.3 eV) and between O 2p and Ti orbitals (in the range of −7.2 to −6.5 eV), suggesting strong chemical bonds between them [30, 31], which may account for the stable retention of the hex‐O configuration. It is deemed that the incorporation of Cu elements in the alloy via the L‐PBF process not only harnesses the aggregation of Cu atoms at the interface, the feature of which retards the segregation of oxygen atoms, but also gives rise to the co‐distributed enrichment of Cu and O atoms in α/α′‐lath. Especially, as fourth and fifth neighbors, the Cu atoms can form strong attractive electronic interaction and hence strong chemical bonds with hex‐O, accounting for the formation and stabilization of hex‐O, which is difficult to achieve in Ti alloys fabricated by traditional casting with slow cooling rate. Furthermore, the Cu introduction reduces the migration barrier of oct‐O to hex‐O from 2.26 to 1.60 eV (Figure S4). These synergistic electronic and energetic modifications establish the mechanisms for manipulating interstitial oxygen sites in L‐PBFed Ti‐0.40O‐1.60Cu alloy. Detailed calculation results and discussions are shown in the Atomistic Modeling section and Supplementary text 1.

### 2.2 Mechanical Properties

**FIGURE 3:**
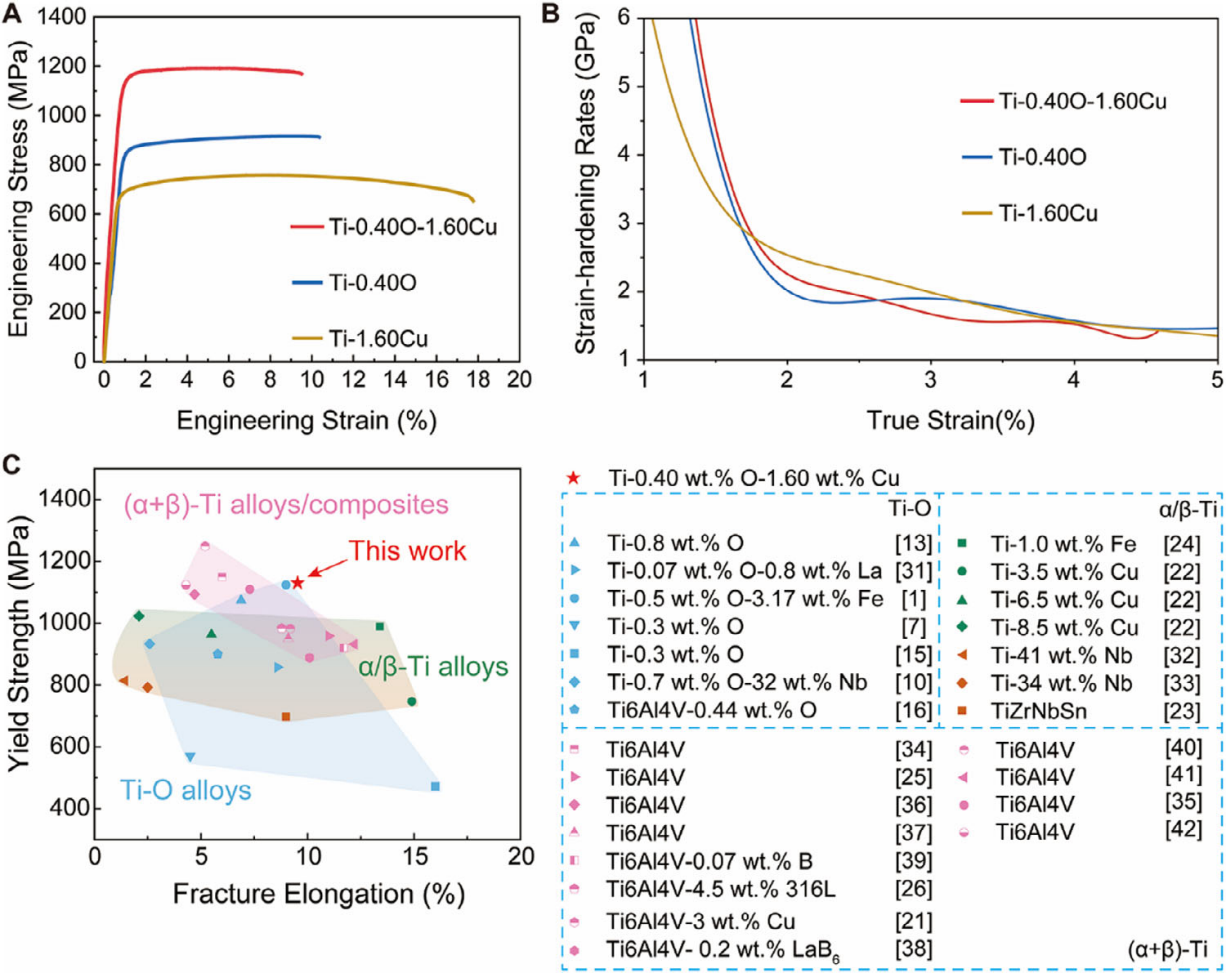
Mechanical properties of the Ti‐0.40O‐1.60Cu, Ti‐0.40O and Ti‐1.60Cu alloys. (A) The tensile engineering stress‐strain curves. (B) The strain‐hardening rates curves. (C) A comparison of the mechanical properties between the present Ti‐0.40O‐1.60Cu alloy and other reported Ti alloys [1, 7, 10, 13, 15, 16, 21, 22, 23, 24, 25, 26, 32, 33, 34, 35, 36, 37, 38, 39, 40, 41, 42, 43].

Strategic manipulation of oxygen interstitial sites through Cu alloying yields markedly improved mechanical properties of the as‐fabricated Ti alloys. As illustrated in Figure S5, for the Ti─O series alloys, the yield strength (YS) and ultimate tensile strength (UTS) gradually increases to 906 MPa and 938 MPa, respectively, as the O content is raised from 0.30 to 0.40 wt.%, accompanied by a significant reduction in fracture elongation (FE). Specifically, the Ti‐0.40O alloy, wherein O atom occupies octahedral sites, exhibits YS and UTS of approximately 906 and 914 MPa, with a FE of 10.4%. Further increasing the O content to 0.45 wt.% results in a Ti‐0.45O alloy with UTS of 1013 MPa and FE of only 3.0%. Additionally, the Ti‐Cu alloy system demonstrates a relatively modest improvement in UTS with Cu content increasing from 1.2% to 1.8 wt.%, with an overall enhancement of approximately 143 MPa. This suggestes a lower strengthening efficiency. By strategically positioning O atom into hexahedral interstitial sites, however, the Ti‐0.40O‐1.60Cu alloy achieves an exceptional strength‐ductility synergy, demonstrating a YS of 1121 MPa, a UTS of 1194 MPa with a FE of 10.2% (Figure 3A). Notably, when the nominal oxygen content is further increased to 0.45 wt.%, the Ti‐0.45O‐1.80Cu alloy demonstrates a 24.3% increase in UTS relative to Ti‐0.45O alloy, while maintaining a slightly higher UE (∼4%) compared to the Ti‐0.45O alloy (∼3%) (Figure S6 and Table S3). This suggests that the crucial role of hex‐O in sustaining uniform deformation becomes more pronounced with higher oxygen content. Figure 3B presents the strain‐hardening behaviors of Ti‐0.40O‐1.60Cu, Ti‐0.40O and Ti‐1.60Cu alloys. It is evident that the Ti‐0.40O‐1.60Cu alloy displays a silghtly higher nominal strain hardening rate than the Ti‐0.40O alloy, particularly prior to reaching a true strain of approximately 2.5%. Such mechanical performance surpasses that of the majority of previously reported Ti‐O alloys (blue dots) [1, 7, 10, 13, 15, 16, 32], and is also competitive, compared with partial α‐Ti alloys (green dots) [22, 24], β‐Ti alloys (orange dots) [23, 33, 34], as well as (α+β)‐Ti alloys/composites (pink dots) [21, 25, 26, 35, 36, 37, 38, 39, 40, 41, 42, 43] fabricated using AM, as shown in Figure 3C. Notably, such exceptional mechanical performance is achieved by adding a moderate amount of Cu element, instead of relaying on the incorporation of substantial amounts of alloying elements typically present in traditional high‐strength titanium alloy. Moreover, the process does not necessitate energy‐intensive or time‐consuming post‐processing techniques, including hot isostatic pressing (HIP) or complex heat treatments. These characteristics underscore the cost‐effectiveness and compatibility with industrial manufacturing processes.

### 2.3 Deformation Mechanisms Dominated by Hexahedral Interstitial Oxygen

**FIGURE 4:**
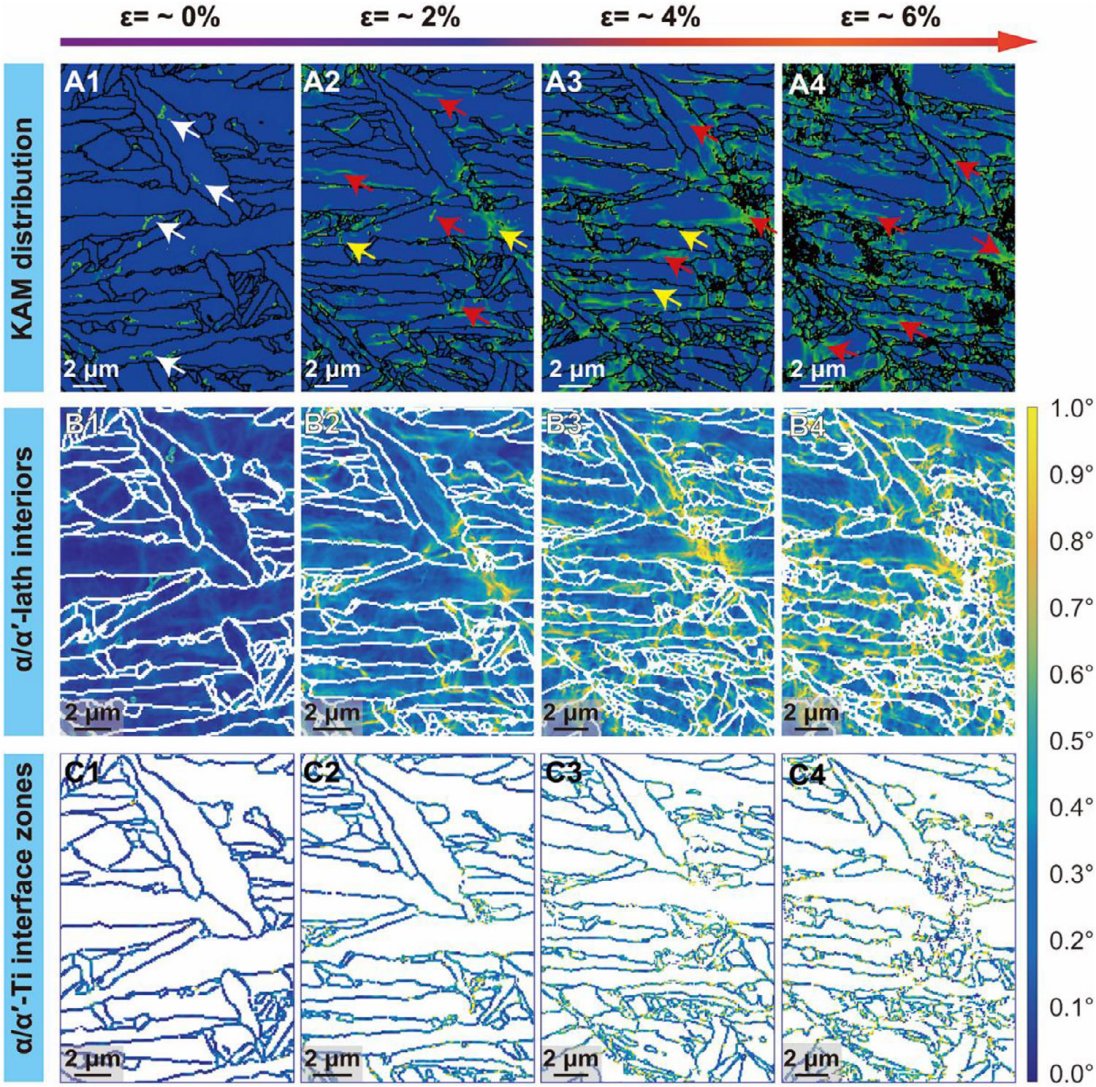
Microstructure evolutions under the in situ EBSD experiment of the Ti‐0.40O‐1.60Cu alloy during uniaxial tension. (A1–A4) KAM diagrams under different strains. (A1) ∼0% strain. (A2) ∼2% strain. (A3) ∼4% strain. (A4) ∼6% strain. The KAM evolution of the (B1–B4) α/α′‐lath interiors and (C1‐C4) α/α′‐lath interface zones.

In situ EBSD analysis performed during uniaxial tension elucidates the deformation mechanisms responsible for the enhanced mechanical performance of Ti‐0.40O alloy (Figure S7) and Ti‐0.40O‐1.60Cu alloy (Figure 4). The hot spots (indicated by white arrows) of kernel average misorientation (KAM) observed in the undeformed Ti‐0.40O alloy (Figure S7A1) and Ti‐0.40O‐1.60Cu alloy (Figure 4A1) are attributed to initial lattice distortion induced by interstitial O or substitutional Cu atoms within the Ti matrix, or potentially to residual internal stresses arising from the AM process. To elucidate the evolution of heterogeneous KAM distribution and its correlation with dislocation density during mechanical straining [44, 45], the deformed microstructure of the Ti‐0.40O–1.60Cu and Ti‐0.40O alloy was partitioned into two distinct regions: the α/α′‐lath interiors (Figure 4B1–B4; Figure S7B1–B4) and α/α′‐lath interface zones (Figure 4C1–C4; Figure S7C1–C4). The corresponding statistical results of KAM value and geometrically necessary dislocation (GND) density are displayed in Figure S8 and Table S4.

KAM distribution (Figure S7) and statistical results (Figure S8) demonstrate that the interaction between oct‐O and dislocations in the Ti‐0.40O alloy leads to the accumulation of dislocations within the α/α′‐lath during the initial strain deformation. However, as the strain increases, particularly beyond ∼4%, the GND density and KAM value at α/α′‐lath interface zones exceed those observed in the α/α′‐lath interiors (Figure S8). This transition may be attributed to the migration of oct‐O during plastic deformation, which prevents it from acting as a stable obstacle to dislocation motion or from consistently activating new dislocation sources [15, 20]. As a result, the interactions between α/α′‐lath interface and dislocation becomes more prominent in the deformation behavior of the Ti‐0.40O alloy.

However, Ti‐0.40O‐1.60Cu alloy exhibits an opposite trend in KAM evolution, indicating distinct deformation mechanisms. Specifically, as deformation progresses, the localization of plastic strain initially observed at the α/α′ lath interfaces (indicated by yellow arrows) gradually transitions to the lath interiors (red arrows) (Figure 4A2–A4). The statistical results in Figure S8 further shown that as strain increases, the α/α′‐lath interior exhibits a significantly higher growth rate in both GND density and KAM than the α/α′‐Ti interface zone. This phenomen is speculated to be driven by the dynamic intersection between hex‐O and dislocations, where hex‐O could act as stable obstacles to dislocation glide. The evidence from first‐principles calculations (Figure S9) and TEM characterization (Figure S10) is provided and discussed in the next section. This persistent positioning of hex‐O enables sustained interaction with dislocation cores, acting as effective pinning sites. As dislocations propagate through the α/α′‐lath interiors, they repeatedly encounter and escape from these stable hex‐O sites, which not only sustains dislocation mobility but also stimulates dislocation multiplication and entanglement. As a result, the GND density in the α/α′‐lath interior of Ti‐0.40O‐1.60Cu keeps increasing with strain and reaches ∼7.20 × 10^14^ m^−2^ at ∼6% strain, even slightly higher than that of the α/α′‐lath interface zones. Such intragranular accumulation of dislocations can efficiently accommodate the plastic strain, instead of relying mainly on dislocation pile‐up near the α/α′‐lath interface [44, 46]. This facilitates a more homogeneous strain distribution across α/α′‐lathes, thereby delaying exhaustion of local deformability. The aforementioned findings reveal that the dislocation accumulation are initially accommodated by the α/α′‐lath interface before being harnessed by hex‐O‐activated α/α′‐lath interiors at early deformation. With increased straining, the hex‐O not only enhances the dislocation activity within the α/α′‐lath interior but also modulates the spatial distribution of KAM between α/α′‐lath interior and interface regions. Such a dislocation‐dependent plasticity transition induces sequential strain partitioning, which alleviated localized stresses at interfaces and sustains strain hardening via the accumulation of high GND density in interiors.

In addition, ex situ X‐ray diffraction (XRD) analysis was also applied to evaluate the statistical dislocation density in the Ti‐0.40O and Ti‐0.40O‐1.60Cu alloys at various deformation stages: the as‐fabricated condition, approximately 6% strain, and the fractured state (Supplementary text 2). The estimated dislocation densities reveal that although the initial dislocation density of the as‐fabricated Ti‐0.40O‐1.60Cu alloy is slightly lower than that of Ti‐0.40O alloy, it increases significantly more with deformation strain (10.97 × 10^14^ m^−2^ and 4.38 × 10^14^ m^−2^ for the Ti‐0.40O‐1.60Cu and Ti‐0.40O alloys, respectively). This pronounced dislocation multiplication observed in the Ti‐0.40O‐1.60Cu alloy further suggests that hex‐O plays a dominant role in promoting dislocation activity than oct‐O, which contributes to the impressive mechanical properties.

**FIGURE 5:**
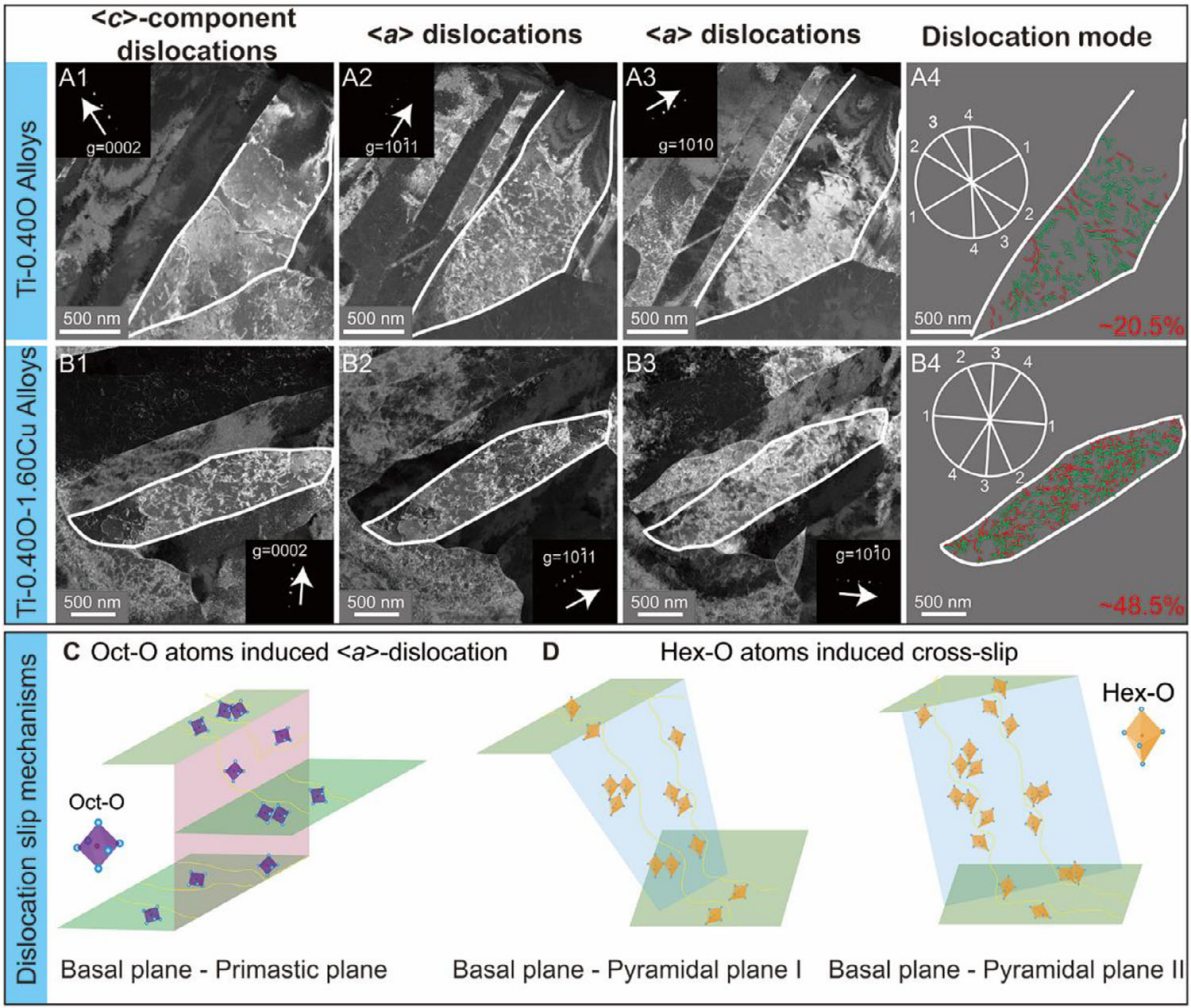
Dislocation configurations analysis of the Ti‐0.40O and Ti‐0.40O‐1.60Cu alloys after plastic deformation. (A1–A3) Ti‐0.40O alloy and (B1–B3) Ti‐0.40O‐1.60Cu alloy showing the < ***c*** >‐component and < ***a*** >‐type dislocations. (A4) and (B4) The schematic diagram of dislocation lines and the content of < ***c*** >‐component dislocation for the Ti‐0.40O and Ti‐0.40O‐1.60Cu alloys, where red and green lines denote < ***c*** >‐component and < ***a*** >‐type dislocations, respectively. The illustration of trace analysis in white circles, where ‘1’ and ‘3’ represent basal and prismatic planes, ‘2’ and ‘4’ represent pyramidal plane I. (C,D) Schematic illustration of interaction between the O interstitial atoms and dislocations. (C) The < ***a*** >‐type dislocation induced by oct‐O on the basal plane {0001} and prismatic plane {$10\bar{1}0$}. (D) The cross‐slip of < ***c*** >‐component dislocation induced by hex‐O on basal plane {0001}, pyramidal plane I {$10\bar{1}1$} and pyramidal plane II {$11\bar{2}2$}. [Correction added on 31 January 2026 after online publication: figure 5 caption is updated.]

The enhanced ductility observed in the Ti‐0.40O‐1.60Cu alloy is attributed not only to dislocation proliferation but, more critically, to the activation of a greater diversity of slip systems. With progressive straining, hex‐O in α/α′‐lath interiors drives a transition in dislocation nucleation regime. The anisotropic strain fields around hex‐O promote the formation of < ***c*** >‐component dislocations rather than < ***a*** > dislocations alone, which exhibit greater mobility across lattice planes and enable strain accommodation in the c‐axis direction, which is unachievable with prismatic < ***a*** > slip. To elucidate the dislocation configuration characteristics, we performed ***g · b*** analysis on the Ti‐0.40O and Ti‐0.40O‐1.60Cu alloys after fracture failure by using double‐beam condition TEM characterizations, as shown in Figure 5. Specifically, in Ti‐0.40O alloy, < ***a*** > dislocations are predominantly observed on both basal and prismatic planes (Figure 5A2 and A3). However, < ***c*** >‐component dislocation, constituting approximately 20.5% of the total, exhibit a planar slip mode (indicated by the red arrow in Figure S11) and glide on the pyramidal plane I. Furthermore, dislocation proliferation occurs primarily through transmission between the basal and prismatic planes. Such dislocation slide mode is illustrated schematically in Figure 5C.

Conversely, the Ti‐0.40O‐1.60Cu alloy demonstrates a significant increase in the proportion of < ***c*** >‐component dislocation on pyramidal plane I and II, reaching approximately 48.5% (Figure 5B1–B4; Figure S12), which satisfying the Von Mises plasticity criteria. Moreover, hex‐O enhances < ***c*** >‐component dislocation multiplication by acting as ‘pinning nodes’ for dislocation segments. The observations of Ti‐0.40O‐1.60Cu during tensile deformation reveal that < ***c*** >‐component dislocations on pyramidal planes exhibit characteristic bow‐out and zigzag morphologies (Figure 5B1). As these dislocations glide, their cores are repeatedly pinned by the hex‐O complex, which creates localized stress concentrations that trigger the activation of Frank‐Read sources, thereby promoting dislocation multiplication [4, 47, 48, 49]. Notably, the activation of dislocation locks at the intersection of the slip system between the basal plane and pyramidal plane I and II provides additional and independent slip systems that facilitate plastic deformation, as elucidated in Figure 5D. Importantly, these locks formed by the interaction of hex‐O‐stabilized < ***c*** >‐component dislocation with < ***a*** > dislocations further enhance strain hardening by impeding dislocation glide, while the diverse slip systems enabled by < ***c*** >‐component dislocations ensure homogeneous strain distribution across α‐laths, avoiding the localized necking that plagues Ti‐0.40O alloy.

First‐principles calculations provide a rationale for the observed hex‐O‐assisted < ***c*** >‐component dislocation activity. Atomic structure models, depicted in Figure S13, were constructed allowing unrestricted movement of both hex‐O and oct‐O configurations. Our calculations demonstrate that hex‐O configurations exhibit lower generalized stacking fault (GSF) energy on pyramidal I and II planes compared to both oct‐O structures and pure Ti (Figure S9). This reduction in GSF suggests that the activation of < ***c*** >‐component dislocation is significantly promoted via short‐range interactions between hex‐O atoms and dislocation cores [4]. Additionally, we further compared the interstitial oxygen atom positions in deformed Ti‐0.40O and Ti‐0.40O‐1.60Cu alloys. As shown in the Figure S14, most of the original oct‐O sites in the Ti‐0.40O alloy have transitioned to hex‐O sites. This interstitial shuffling mechanism, previously reported in simulation studies, is considered to induce a slip plane softening effect, promoting planar slip behavior [14, 20]. In contrast, hex‐O atoms in the deformed Ti‐0.40O‐1.60Cu alloy remain at their initial positions without migration (Figure S10C,D). These hex‐O induced distortions in the arrangement of Ti matrix atoms within the dislocation core region (Figure S10E). Such pronounced repulsive effect, extending beyond the dislocation cores [15], effectively pins dislocations in a continuous manner. These atomic‐scale mechanisms collectively explain the enhanced < ***c*** >‐component dislocation activity while maintaining effective strain hardening capacity in the Ti‐0.40O‐1.60Cu alloy.

## 3 Conclusion

To summarize, this work presents a strategy to overcome the inherent strength‐ductility trade‐off in oxygen‐strengthened titanium alloys by regulating oxygen interstitial positions. Utilizing L‐PBF processing in conjunction with Cu─O co‐alloying, we successfully stabilized hex‐O configurations within a Ti‐0.40O‐1.60Cu alloy. Atomic‐scale characterization and theoretical calculations reveal that Cu incorporation modifies the local electronic structure, effectively reducing the energy barrier for oxygen transitions from octahedral to hexahedral sites while preserving thermodynamic stability. This hex‐O configuration concurrently activates multiple slip systems and maintains effective dislocation pinning via long‐range repulsion, ultimately yielding a YS of 1121 MPa and a FE of 10.2%. This interstitial site control strategy offers a promising avenue for designing high‐performance titanium alloys by overcoming the conventional limitations associated with oxygen embrittlement.

## 4 Materials and Methods

### 4.1 Materials

The feedstock powders utilized in this research consist of commercial pure Ti powder (15∼53 µm, Nantong IMT Company), pure Cu powder (∼10 µm, ZKKY Company) and high purity (99.5%) CuO particles and TiO_2_ particles (∼10 µm, Shanghai Macklin Biochemical Technology Co., Ltd.). The weight ratios of the feedstock were determined based on the designed alloy compositions: Ti‐ (0.5 wt.%–2.25 wt.%) CuO, Ti‐ (0.874–1.248) wt.% TiO_2_, Ti‐ (1.4–3) wt.% Cu and pure‐Ti. The determined powders were mixed with alumina ceramic balls (diameter: 3 mm) in a 3D mixer at a ball‐to‐powder ratio of 1:1, with a rotation speed of 50 rpm for 16 h. The morphologies of the Ti‐CuO composite powders were shown in Figure S15. Subsequently, the powders were preheated at 100°C for 1 h under vacuum conditions and then cooled to room temperature. The bulk and tensile samples were fabricated by EP‐M260 (Laser Powder Bed Fusion, L‐PBF, Eplus3D, Beijing) under an argon atmosphere (line spacing: 120 µm, layer thickness: 30 µm, scanning speed: 800 mm/s, and laser power: 180 W). All the samples were prepared following the same procedure. The nominal and measured contents of O and Cu in Ti powders and Ti‐O‐Cu bulk samples are summarized in Table S5.

### 4.2 Mechanical Testing

The AGS‐X universal tensile testing machine, equipped with RVX‐112B video extensometer, was utilized for tensile testing at a loading rate of 10^−3^ mm/min under room temperature. The tensile specimens featured a gauge length of 20 mm, with a scale distance measuring 8 mm in length and 1 mm in thickness.

### 4.3 Microstructural Characterization

The morphologies of the powders were examined by the field emission scanning electron microscopy (SEM, Hitachi S‐4800). The high‐resolution JEM‐F200 transmission electron microscopies (TEM) at 200 kV with energy‐dispersive spectroscopy (EDS) were used to characterize the morphology and element distribution. FEI Talos F200X G2 transmission electron microscopy (TEM) at 200 kV was utilized to investigate dislocation evolution. The type of dislocation was identified based on the invisible criterion of ***g*** · ***b*** = 0, where ***g*** is the diffraction vector and ***b*** denotes the Burgers vector. According to the diffraction pattern reported in the literature [50], the trace analysis results are presented in Figure 5A4 and Figure 5B4 and Figure S12D. The atomic structure analysis was performed by a double aberration‐corrected FEI Titan‐Themis‐Z TEM at 300 kV with high‐angle annular dark‐field scanning TEM (HAADF‐STEM) mode. The acquired data were subsequently analyzed using Digital Micrograph 3.4.

Electron backscatter diffraction (EBSD) was conducted by a Thermo Scientific Apreo 2C‐Scaning Electron Microscope (SEM) equipped with an EDAX Velocity Super, with an acceleration voltage of 20 kV and a step size of 80 nm. The EBSD data were analyzed using both the channel 5 software and the open‐source MATLAB toolbox MTEX. Given that the pronounced disparity of the kernel average misorientation (KAM) between within 100 nm of α/α′‐lath interface zones compared to those deeper within the α/α′‐lath interiors, a threshold of 100 nm was established for the minimum Euclidean distance in this study [44]. The geometrically necessary dislocation (GND) density (ρ_*GND*_) can be calculated based on the KAM values as follows [51]:

(1) $$\rho_{GND}=\frac{2\theta }{\mu b}$$

where θ is the KAM value, μ is the step size (80 nm) and *b* is the Burges vector of α‐Ti matrix (0.295 nm).

Atom probe tomography (APT) experiments were conducted on a CAMECA LEAP 4000X Si instrument under the following conditions: laser energy of 80 pJ, pulse repetition rate of 200 kHz, specimen temperature of 40 K and detection rate of 0.5%. The corresponding data were reconstructed and analyzed through the IVAS 3.6.8 software package provided by CAMECA. APT sharp needles were prepared using a Zeiss crossbeam SEM/FIB system to perform standard lift‐out procedure from site‐specific regions of mechanical polished bulk Ti alloy samples.

X‐ray diffraction (XRD) experiments were conducted by Rigaku Smartlab SE with Cu Kα radiation (λ = 1.54056 Å) over 2θ range of 20°–90° with a scanning speed of 2° min^−1^. The micro‐strain was calculated based on the full width at half maximum (FWHM) of the diffraction peaks using the Williamson‐Hall method [52, 53]. The detailed information is provided in Supplementary text 2.

### 4.4 Atomistic Modeling

The atomistic simulation was performed through the Vienna ab initio simulation package based on density functional theory (DFT). The interaction between ions and electrons was described using the Projector‐Augmented‐Wave (PAW) method. The exchange‐correlation energy was modeled using the Perdew‐Burke‐Ernzerhof (PBE) functional with the generalized gradient approximation (GGA). A plane wave basis set with a cut‐off energy of 400 eV was employed. The convergence criteria for electronic self‐consistency and ion relaxation were set to 10^−4^ eV and 0.02 eV/Å, respectively.

In order to evaluate the interaction energy between O atoms and Cu atoms at different position, a supercell consisting of 128‐atom Ti atoms and 1‐atom O atom was constructed, where the O atom was inserted into both hexahedral and octahedral sites separately. Ti atoms adjacent to the O atom at various distances were replaced with Cu atoms. The typical atomic models are shown in Figure S16. The binding energy between Cu atom and O atom can be calculated from the following expression:

(2) $$E_{Bind}^{O-Cu}=\left(E^{O-Cu}+E\right)-\left(E^{O}+E^{Cu}\right)$$

where *E* ^*O* − *Cu*^ represents the total energy of the structure containing both O and Cu element. The *E^X^* (X = O, Cu) denotes the total energies of the structure containing only O or Cu atoms, respectively. *E* represents the energy of the pure Ti in original supercell. The negative calculated binding energy indicates an attractive interaction between interstitial O atom and Cu atom. In addition, the energy barrier for the diffusion migration of interstitial O atom from an octahedral to a hexahedral site in the model containing hex‐O and the 5th Cu was calculated. This migration path was analyzed by considering five distinct states: the initial state, three intermediate states and the final state. The minimum energy path (MEP) was determined using the climbing image nudged elastic band (CINEB) method 54. During the optimization process, the path was iteratively refined until the residual forces on all images were reduced below 0.02 eV/Å.

To evaluate the effect of O atoms at different interstitial positions on the slip systems, the generalized stacking fault (GSF) energy (*E_GSF_*) was calculated. Models of a pure Ti matrix with oxygen atoms located in either hexahedral or octahedral sites were established to simulate the GSF of various slip systems, including pyramidal plane I $\left\{10\bar{1}1\right\}\left\langle 11\bar{2}3\right\rangle$ and pyramidal plane II $\left\{11\bar{2}2\right\}\left\langle 11\bar{2}3\right\rangle$. The atomic models reserved a 10 Å vacuum region along the slip direction. The *E_GSF_* was calculated using the following formula:

(3) $$E_{GSF}=\frac{E_{x}-E_{0}}{A}$$

where *x* represents the slip distance, *E_x_* and *E*_0_ represent the total energy after slip and the original structure energy, and *A* is the cross‐sectional area.

## Author Contributions

X.L. and X.R. initiated and supervised the project. X.L. prepared materials and conducted mechanical testing. X.L. performed HETEM. L.M. and G.S. performed and process the APT results. S.P. and F.Q. conducted HADDF‐STEM characterizations. X.L. and Z.X. processed HAADF‐STEM data. J.X., J.W. and X.W. performed first‐principles calculation based on DFT. D.Z., X.Z., C.S., C.H. and N.Z. discussed the results. X.L., X.R. and D.Z. wrote the manuscript.

## Conflicts of Interest

The authors declare no conflict of interest.

## Supporting information




**Supporting File 1**: advs74042‐sup‐0001‐SuppMat.docx.

**Supporting File 2**: advs74042‐sup‐0002‐FigureS1‐S19.zip.

## Data Availability

The data that support the findings of this study are available in the supplementary material of this article.
