# Supplementary material for: Addressing Oxygen Embrittlement in Additively Manufactured Titanium via Cu‐Mediated Interstitial Site Engineering
Source: Adv Sci (Weinh). 2026 Jan 29;13(19):e19184. doi: 10.1002/advs.202519184 (PMC13045232; doi:10.1002/advs.202519184)
Supplement: Supplementary file 1 — Supporting File 1: advs74042‐sup‐0001‐SuppMat.docx. [file ADVS-13-e19184-s002.docx]

***Supplementary Materials for***

Addressing oxygen embrittlement in additively manufactured titanium via Cu-mediated interstitial site engineering

*Xiaobin Lin* *^a^, Xudong Rong ^a, b *^, Jiachen Xie ^a^, Xinru Wang ^a^, Jianteng Wang ^a^, Zhihang Xu ^a^, Dongdong Zhao ^a, c *^, Shiwei Pan ^d^, Feng Qian ^d^, Longlong Ma ^e^, Gang Sha ^e^, Xiang Zhang ^a, b^, Chunsheng Shi ^a^,Chunnian He ^a, c^, Naiqin Zhao ^a, b *^*

*^a^ Tianjin Key Laboratory of Composite and Functional Materials, School of Materials Science and Engineering, Tianjin University, Tianjin 300350, People’s Republic of China*

*^b^ State Key Laboratory of High Performance Roll Materials and Composite Forming, Tianjin University, Tianjin 300350, People’s Republic of China*

*^c^ State Key Laboratory of Precious Metal Functional Materials, Tianjin University, Tianjin 300350, People’s Republic of China*

*^d^ National Key laboratory of Science and Technology on Materials Under Shock and Impact, School of Materials Science and Engineering, Beijing Institute of Technology, Beijing, 100081, People’s Republic of China*

*^e^* *Herbert Gleiter Institute of Nanoscience, School of Materials Science and Engineering, Nanjing University of Science and Technology, Nanjing, People’s Republic of China*

*^∗^Corresponding authors.*

*E-mail addresses: ddzhao@tju.edu.cn, xdrong@tju.edu.cn, nqzhao@tju.edu.cn*


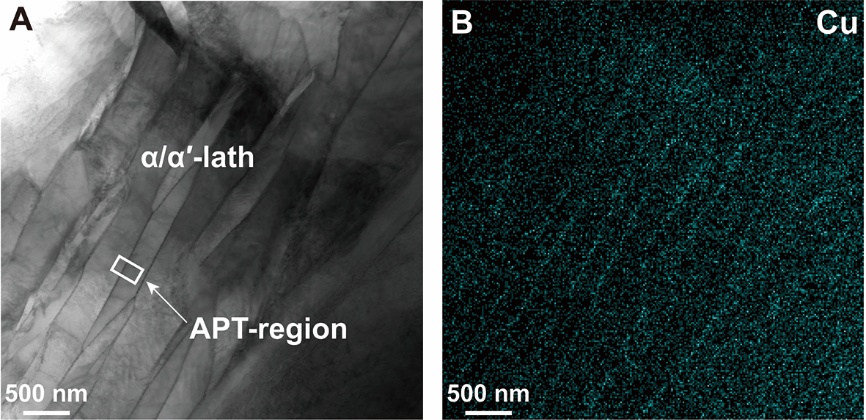


**Figure. S1.** The TEM images of the Ti-0.40O-1.60Cu alloy fabricated by L-PBF. (A) The lamellar α/α′-lath where the white rectangular area is selected for APT characterization. (B) The energy-dispersive spectroscopy (EDS) mapping showing the Cu element distribution.


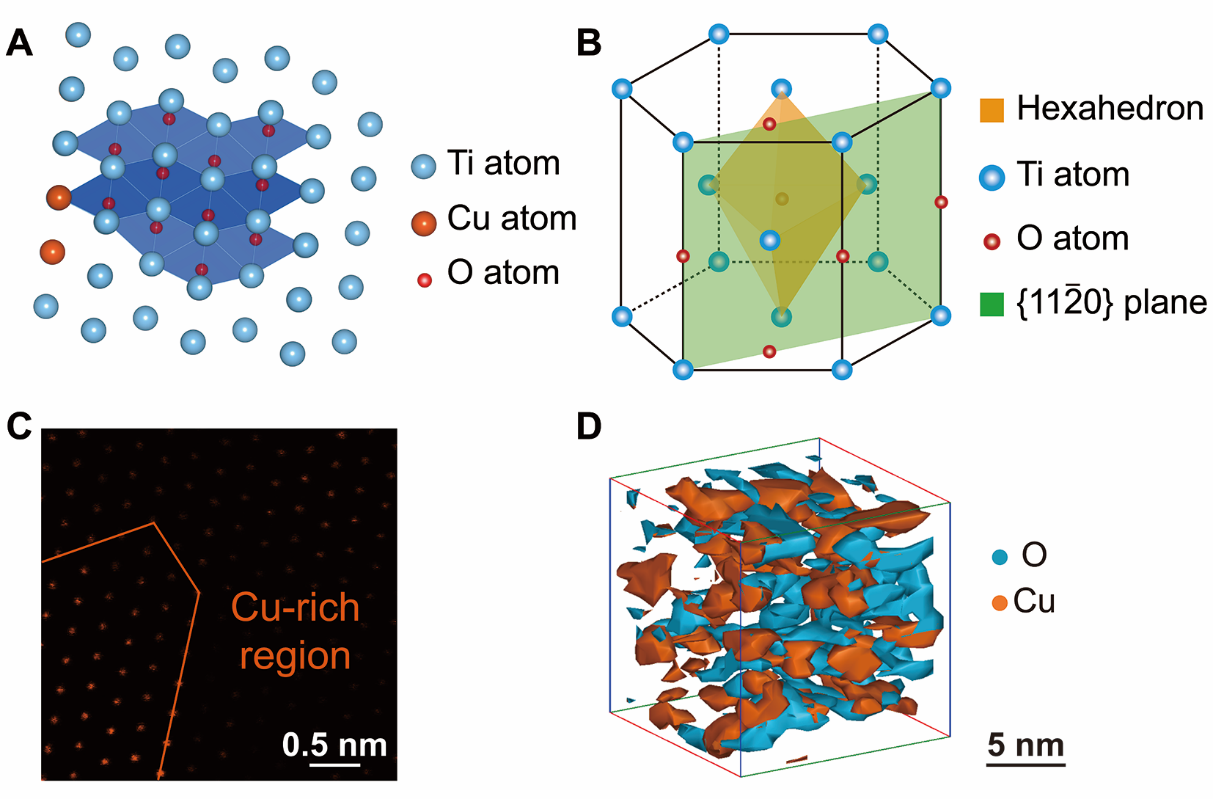


**Figure. S2.** (A) The crystal atomic model. (B) Schematic diagrams of hexahedral interstitial site space on the $\{11\bar{2}0$} plane. (C) The Cu distribution with different uneven Z-contrast according to Fig. 2B in main text. (D) The partial enlarged view in Fig. 1F in main text with the 2.0 at.% iso-composition surface of O atoms (blue) and the 1.5 at.% iso-composition surface of Cu atoms (orange) in α/α′-lath.


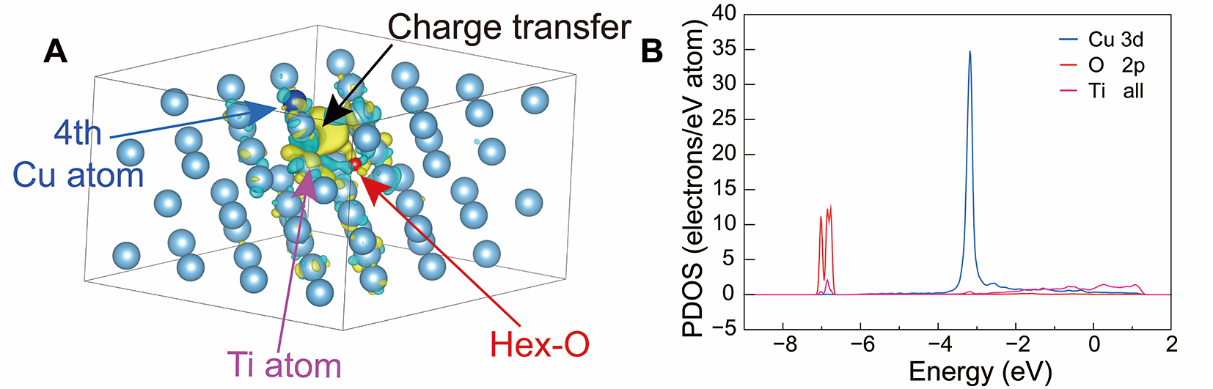


**Figure. S3.** (A) The interfacial difference charge density of Ti-0.40O-1.60Cu alloy with 4th Cu atom at iso-surface level of 1.50×10^-4^ e/Bohr^3^. (B) The partial density of states (PDOS) calculation results according to the Fig. 2I in main text.


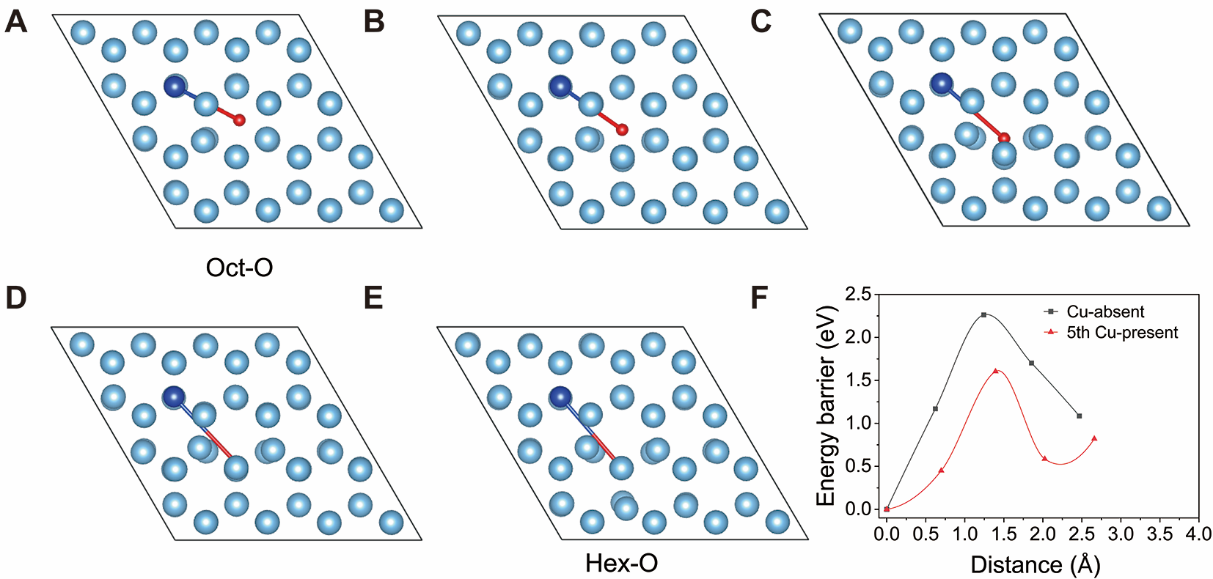


**Figure. S4.** The energy barrier of O atom migration from octahedral site to hexahedral site with/without Cu atom. (A) Oct-O (the red atom) in the Ti matrix (blue atoms) with one Cu atom (navy blue atom). (B) ~ (D) Different stages of O atom migration from octahedral site to hexahedral site. (E) Hex-O (the red atom) in the Ti matrix. (F) The calculated results of energy barrier.


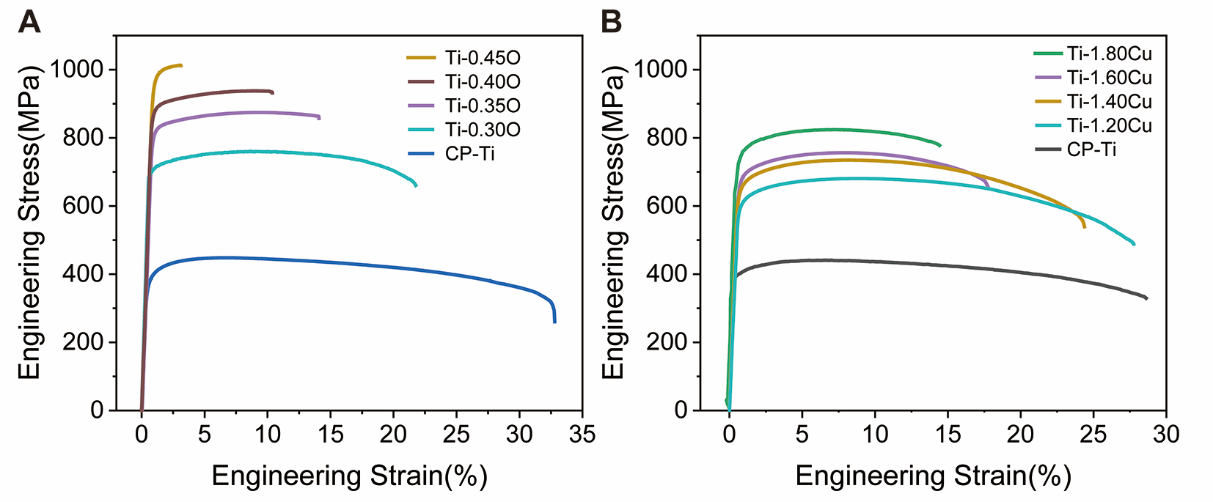


**Figure. S5.** The mechanical properties of the (A) Ti-O alloys and (B) Ti-Cu alloys with different contents of Cu and O atoms.


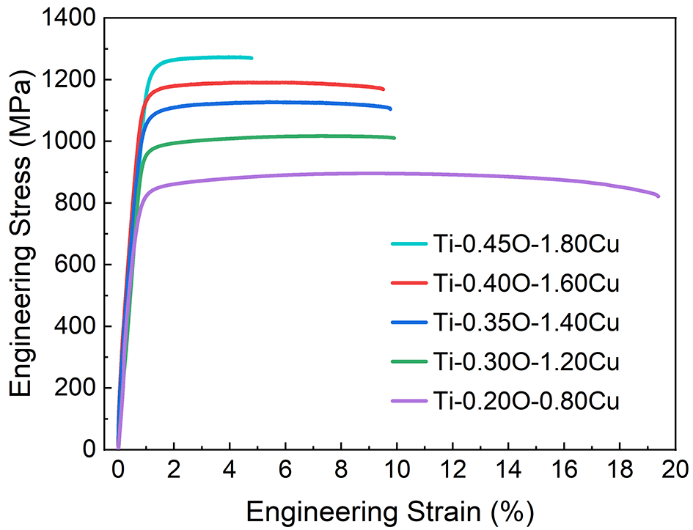


**Figure. S6.** Mechanical properties of Ti-O-Cu alloys with different contents of Cu and O atoms.


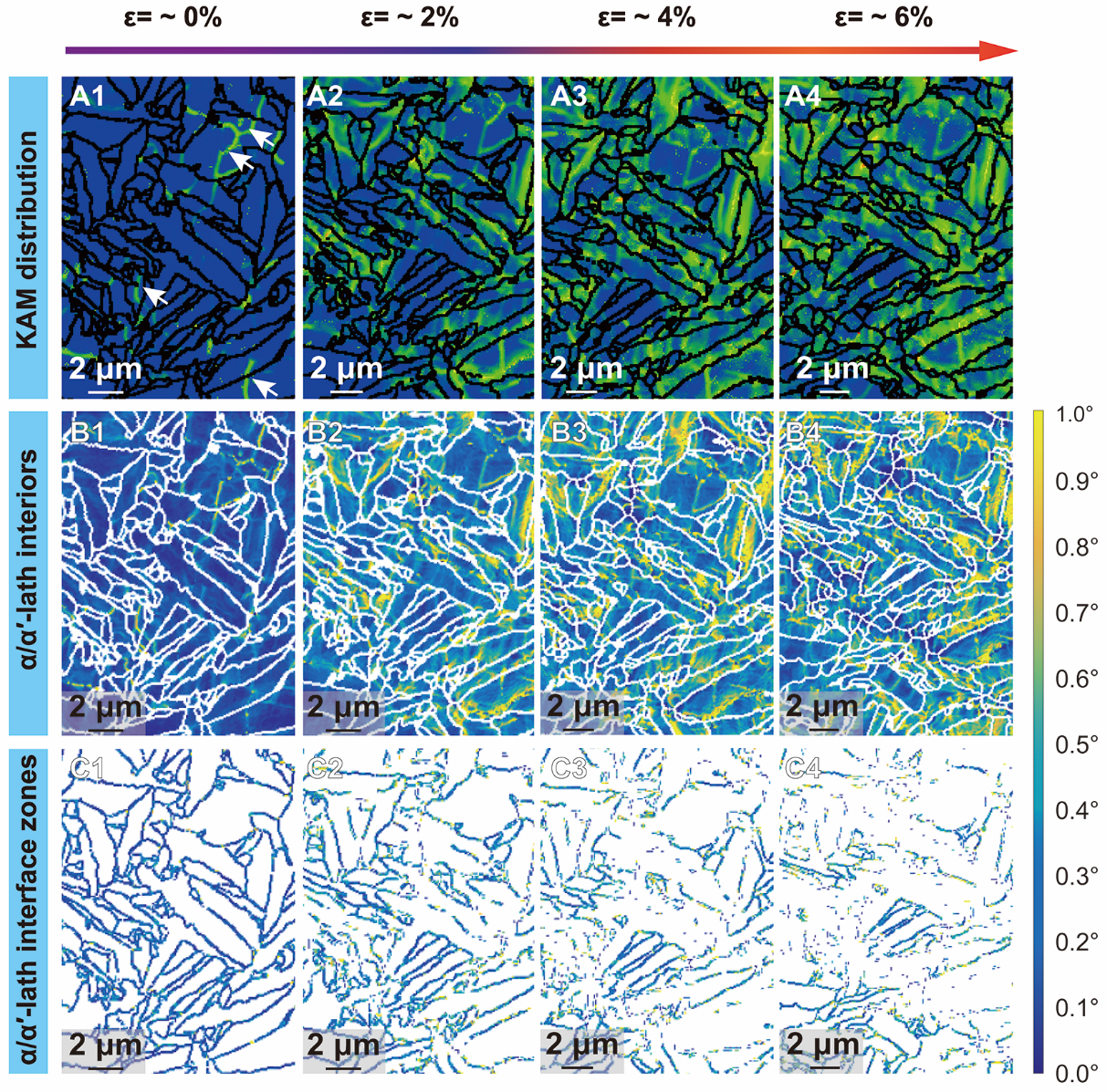


**Figure. S7.** Microstructure evolutions under the in-situ EBSD experiment of the Ti-0.40O alloy during uniaxial tension. (A1)~(A4) KAM diagrams under different strains. (A1) ~0% strain. (A2) ~2% strain. (A3) ~4% strain. (A4) ~6% strain. The KAM evolution of the (B1)~(B4) α/α′-lath interiors and (C1)~(C4) α/α′-lath interface zones.


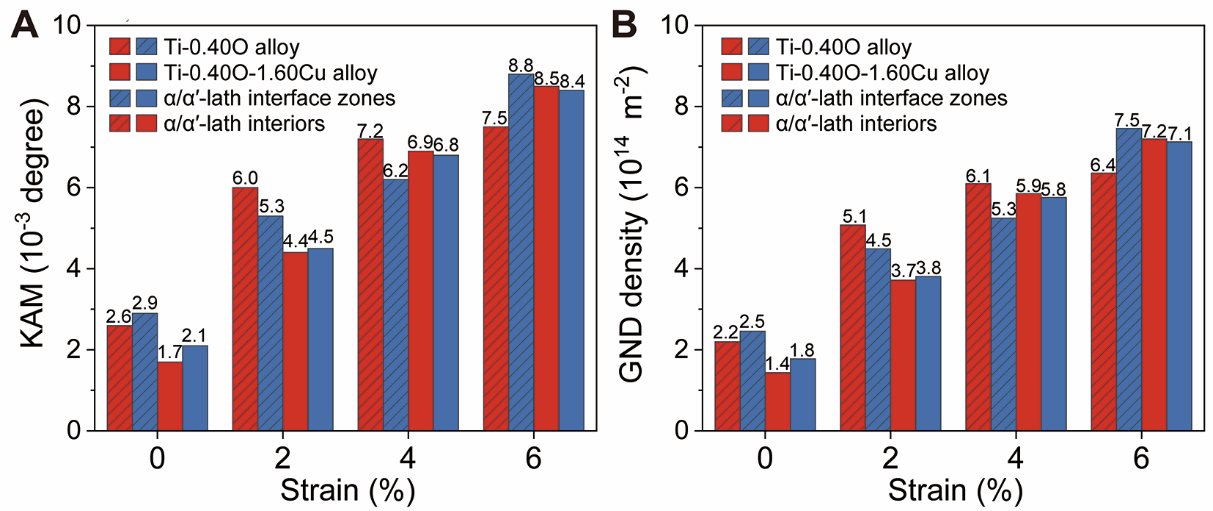


**Figure. S8.** The statistical results of KAM and GND of Ti-0.40O and Ti-0.40O-1.60Cu alloys. The average values of (A) KAM and (B) GND in the α/α′-lath interface zones (blue) and α/α′-lath interiors (red) at different strains.

**
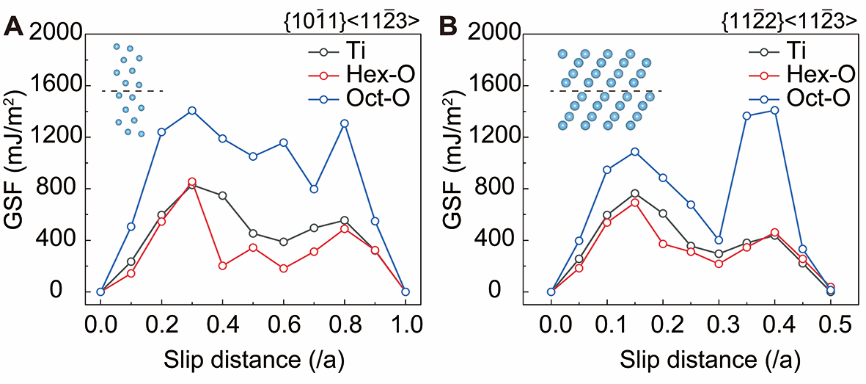
**

**Figure. S9.** The calculation results of GSF in pure-Ti, Ti with oct-O and Ti with hex-O. (A) Pyramidal plane I {$10\bar{1}1$}<$11\bar{2}3$>. (B) Pyramidal plane II {$11\bar{2}2$}<$11\bar{2}3$>.


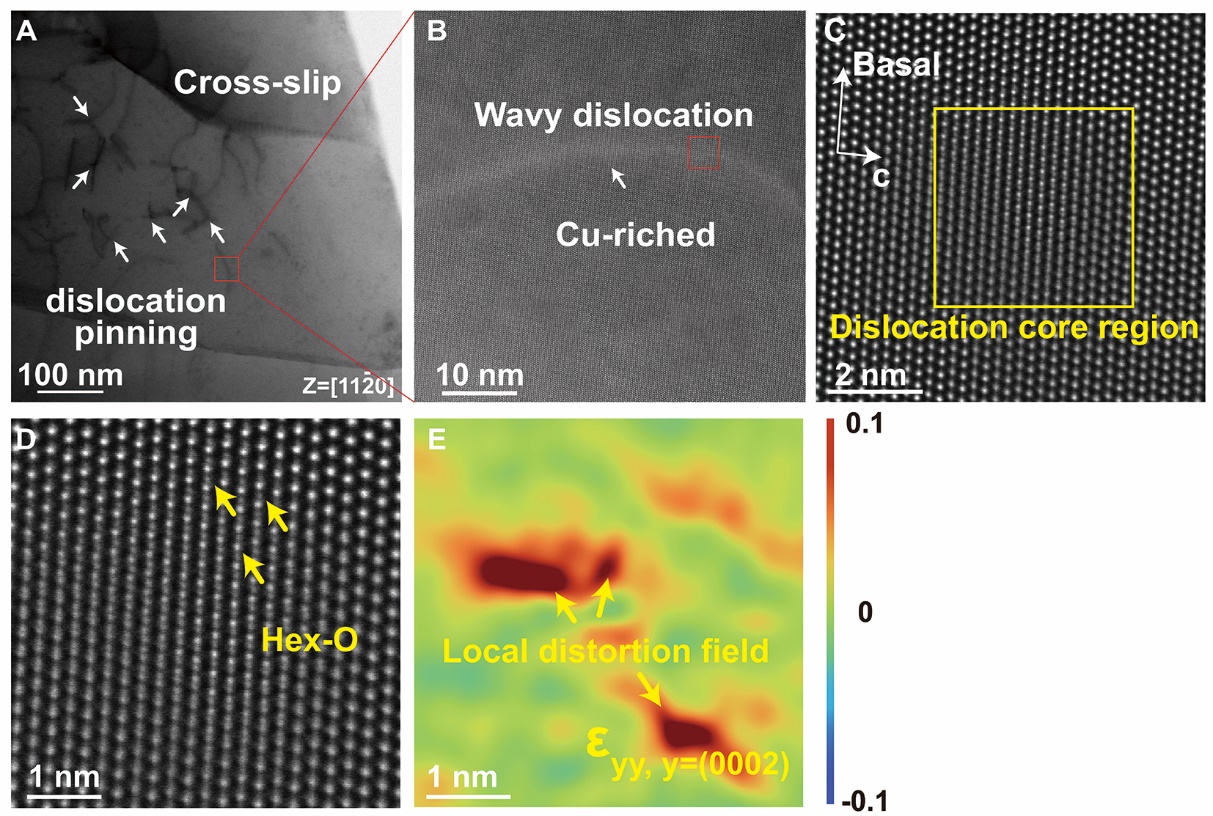


**Figure. S10**. (A) Typical dislocation morphologies in the deformed Ti-0.40O-1.60Cu alloys. (B) The wavy dislocation in the red box of (A). (C) The dislocation core region. (D) The HAADF-STEM image of the enlarged vies of the yellow frame in (C) showing the O atoms located in the hexahedral interstitial sites without migration. (E) The strain distribution map calculated from (C).


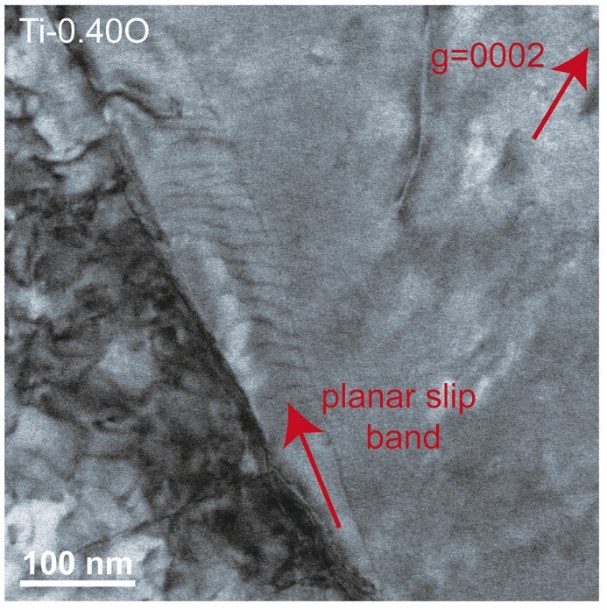


**Figure. S11.** The planar slip band in the deformed Ti-0.40O alloy.


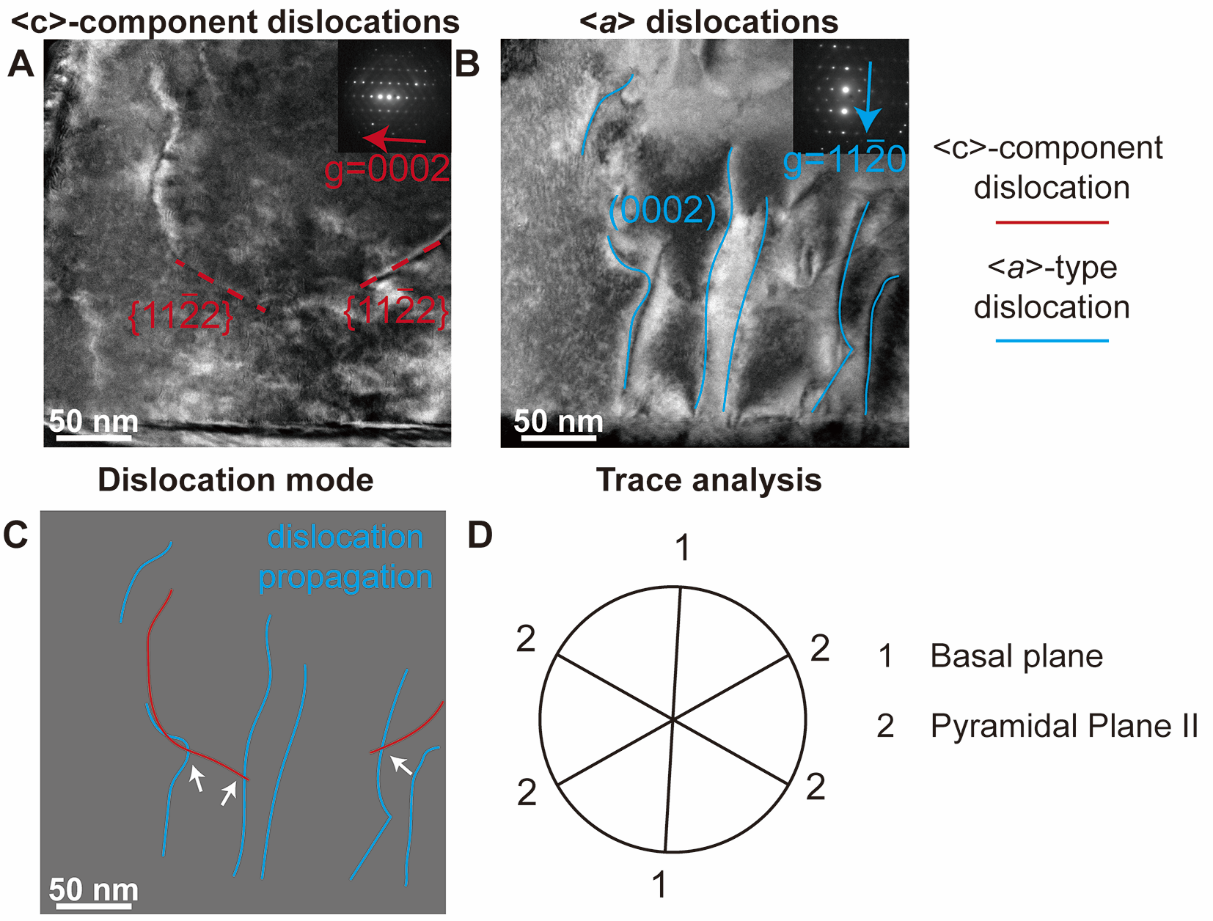


**Figure. S12.** Dislocation configurations analysis of the Ti-0.40O-1.60Cu alloys after plastic deformation under the zone axis [$10\bar{1}0]$. The morphologies of the (A) <*c*>-component dislocation under g=0002 and (B) the <*a*> dislocation under g=$11\bar{2}0$. (C) The schematic diagram of dislocation lines and the content of <*c*>-component dislocation. (D) The corresponding trace analysis.

**
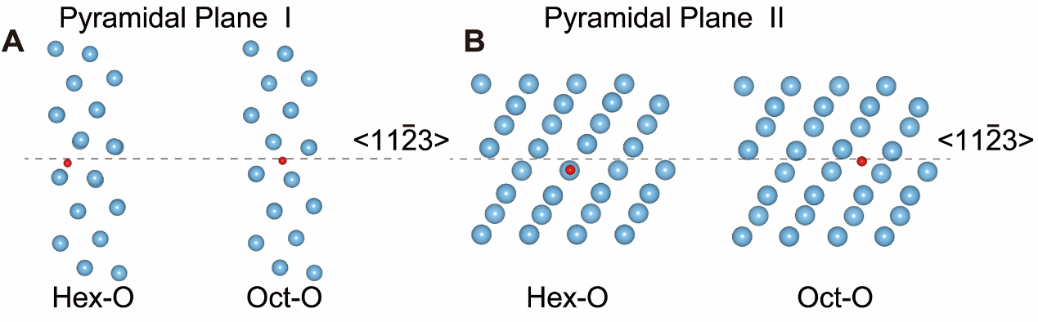
**

**Figure. S13.** Atomic models of hex-O and oct-O for generalized stacking fault (GSF) calculation in various slip systems. (A) Pyramidal plane I {$10\bar{1}1$}<$11\bar{2}3$>. (B) Pyramidal plane II {$11\bar{2}2$}<$11\bar{2}3$>.

**
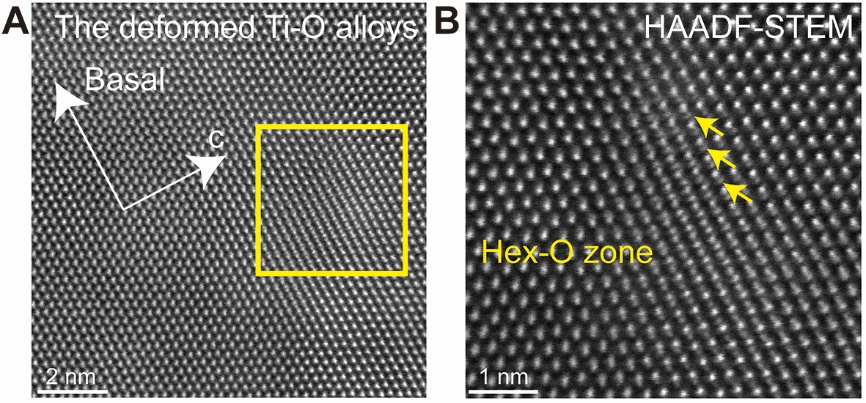
**

**Figure. S14.** (A) The HAADF-STEM characterization in the α/α′-lath of the deformed Ti-0.40O alloys. (B) The enlarged view of the yellow frame of the (A).


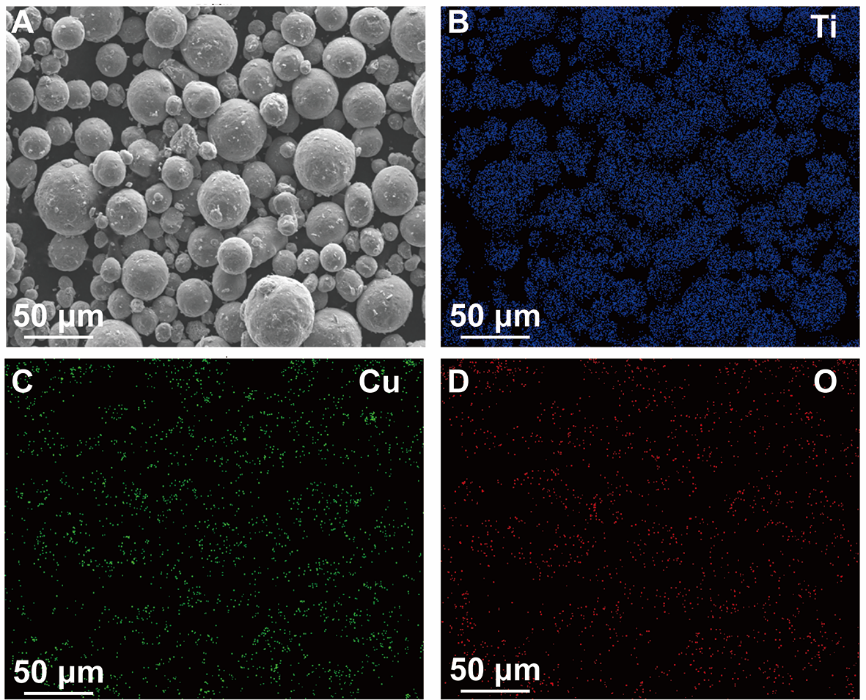


**Figure. S15.** The morphologies of Ti-CuO composite powders prepared for AM. (A) SEM map of the mixed Ti-CuO composite powders. (B)~(D) EDS maps of the Ti, Cu and O elements according to (A).

**
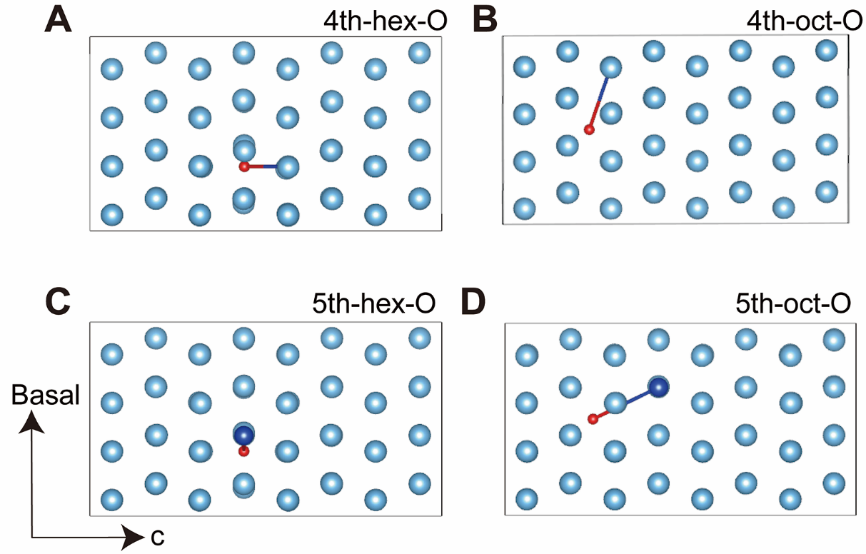
**

**Figure. S16.** The atomic models of the calculation of interaction energy between hex-O/oct-O and Cu atom. (A) 4th-nearest-Cu and hex-O. (B) 4th-nearest-Cu and oct-O. (C) 5th-nearest-Cu and hex-O. (D) 5th-nearest-Cu and oct-O.

**Table S1.** The average length, width and aspect ratio of α-phase in the Ti-0.40O-1.60Cu, Ti-0.40O, Ti-1.60Cu alloys and pure-Ti, respectively.

| Sample | Length (μm) | Width (μm) | Aspect ratio |
| --- | --- | --- | --- |
| Ti-0.40O-1.60Cu | 2.78 ± 0.05 | 0.90 ± 0.01 | 2.91 |
| Ti-0.40O | 3.45 ± 0.09 | 1.03 ± 0.02 | 3.14 |
| Ti-1.60Cu | 3.66 ± 0.10 | 1.21 ± 0.02 | 2.96 |
| Pure-Ti | 13.02 ± 0.63 | 6.48 ± 0.32 | 2.14 |

**Table S2.** The calculation results of interaction energy between Cu atoms, hex-O and oct-O atoms, where ‘+’ represents repulsion and ‘-’ represents attraction.

| Site | Interaction energy (oct-O, eV) | Distance (Å) | Interaction energy (hex-O, eV) | Distance (Å) |
| --- | --- | --- | --- | --- |
| 1st-Cu | 0.620 | 2.08 | 0.400 | 1.92 |
| 2nd-Cu | -0.064 | 3.60 | 0.042 | 2.21 |
| 3rd-Cu | -0.075 | 3.87 | 0.032 | 3.41 |
| 4th-Cu | -0.027 | 4.63 | -0.077 | 3.76 |
| 5th-Cu | -0.085 | 4.86 | -0.110 | 4.54 |
| 6th-Cu | -0.090 | 5.67 | 0.016 | 4.89 |
| 7th-Cu | -0.033 | 6.03 | 0.056 | 5.60 |
| 8th-Cu | -0.049 | 6.70 | -0.018 | 5.76 |
| 9th-Cu | -0.039 | 7.58 | 0.025 | 6.45 |

**Table S3.** The YS, UTS, UE and FE of pure Ti, Ti-O and Ti-O-Cu alloys with different weight contents (wt.%) of Cu and O atoms.

| Sample | YS (MPa) | UTS (MPa) | UE (%) | FE (%) |
| --- | --- | --- | --- | --- |
| Ti-0.45O-1.80Cu | 1177 ± 21 | 1260 ± 9 | 4.0 ± 0.3 | 4.7 ± 0.7 |
| Ti-0.40O-1.60Cu | 1121 ± 17 | 1194 ± 7 | 4.9 ± 0.5 | 10.2 ± 0.7 |
| Ti-0.35O-1.40Cu | 1026 ± 10 | 1116 ± 23 | 5.4 ± 0.3 | 10.0 ± 0.1 |
| Ti-0.30O-1.20Cu | 955 ± 4.6 | 1017 ± 15 | 7.3 ± 0.4 | 9.6 ± 0.3 |
| Ti-0.20O-0.80Cu | 824 ± 8 | 886 ± 7 | 8.9 ± 0.4 | 19.4 ± 0.8 |
| Ti-0.45O | 1005 ± 13 | 1013 ± 8 | 3.0 ± 0.5 | 3.1 ± 0.6 |
| Ti-0.40O | 906 ± 9 | 938 ± 11 | 9.0 ± 0.3 | 10.2 ± 0.8 |
| Ti-0.35O | 842 ± 10 | 874 ± 9 | 8.9 ± 0.3 | 14.1 ± 0.9 |
| Ti-0.30O | 730 ± 6 | 761 ± 5 | 8.7 ± 0.2 | 21.7 ± 0.7 |
| Pure-Ti | 380 ± 6 | 443 ± 3 | 6.6 ± 0.6 | 29.7 ± 0.8 |

**Table S4.** The statistical results of KAM and GND of Ti-0.40O and Ti-0.40O-1.60Cu alloys.

| Sample | Strain | KAM (10^-3^ degree) | | GND density (10^14^ m^-2^) | |
| --- | --- | --- | --- | --- | --- |
|  |  | α/α′-Ti interface zone | α/α′-lath interior | α/α′-Ti interface zone | α/α′-lath interior |
| Ti-0.4O alloy | 0% | 2.9 | 2.6 | 2.46 | 2.2 |
|  | 2% | 5.3 | 6.0 | 4.49 | 5.08 |
|  | 4% | 6.2 | 7.2 | 5.25 | 6.10 |
|  | 6% | 8.8 | 7.5 | 7.46 | 6.36 |
| Ti-0.4O-1.6Cu alloy | 0% | 2.1 | 1.7 | 1.78 | 1.44 |
|  | 2% | 4.5 | 4.4 | 3.81 | 3.73 |
|  | 4% | 6.8 | 6.9 | 5.76 | 5.85 |
|  | 6% | 8.4 | 8.5 | 7.12 | 7.20 |

**Table S5.** The nominal and measured contents of O and Cu in Ti powders and Ti-O-Cu bulk samples.

| Sample | Nominal O  content (wt.%) | Measured O content (wt.%) | Nominal Cu  content (wt.%) | Measured Cu content (wt.%) |
| --- | --- | --- | --- | --- |
| Ti-0.35O-1.40Cu bulk | 0.35 | 0.57 | 1.40 | 1.43 |
| Ti-0.40O-1.60Cu bulk | 0.40 | 0.62 | 1.60 | 1.61 |
| Ti-0.40O bulk | 0.40 | 0.54 | - | - |
| Ti-1.6Cu bulk | - | 0.15 | 1.60 | 1.59 |
| Ti-0.45O-1.80Cu bulk | 0.45 | 0.66 | 1.80 | 1.80 |
| Pure-Ti powder | - | 0.09 | - | - |
| Pure-Ti bulk | - | 0.11 | - | - |

**Supplementary text 1. The DFT calculations of difference charge density transfer between Cu and hex-O.**

To reveal the impact of interaction between Cu atom on O interstitial sites, we conducted difference charge density calculations ($\Delta\rho$) using 64-atom supercells with consistent atomic configurations. The Ti-O-Cu model includes one O atom, one Cu atom, and 63 Ti atoms. The Ti-O model consists of one O atom and 64 Ti atoms. The Ti-Cu model contains one Cu atom and 63 Ti atoms. All oxygen atoms in the models are positioned at the same hexahedral site, while the Cu atom is located at the 5th nearest neighbor position near hex-O. The difference charge density between Cu and O ($\Delta\rho_{Cu-O}$) can be calculated as follows ^[29]^:

$\Delta\rho_{Cu-O}=\Delta\rho_{Ti-O-Cu}-\Delta\rho_{Ti-O}-\Delta\rho_{Ti-Cu}+\Delta\rho_{Ti}$ (S-1)

where $\Delta\rho_{Ti-O-Cu}$, $\Delta\rho_{Ti-O}$, $\Delta\rho_{Ti-Cu}$ and $\Delta\rho_{Ti}$ denote the electron density of the Ti-O-Cu, Ti-O, Ti-Cu and pure Ti models.

**Supplementary text 2. The dislocation densities of Ti-0.40O and Ti-0.40O-1.60Cu samples.**

X-ray diffraction (XRD) was applied to evaluate the statistical dislocation density in the Ti-0.40O and Ti-0.40O-1.60Cu alloys at various deformation stages: the as-fabricated condition, approximately 6% strain, and the fractured state, as shown in Fig. S17. The micro-strain was calculated based on the full width at half maximum (FWHM) of the diffraction peaks using the Williamson-Hall method, with results obtained through linear fitting (Fig. S18) ^[52-53]^. The estimated dislocation densities, presented in Fig. S19.


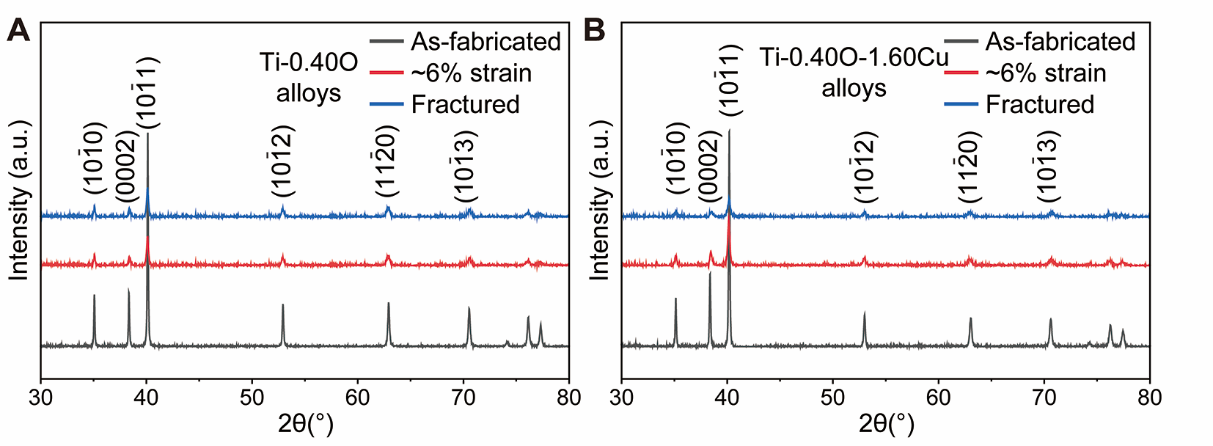


**Figure. S17.** XRD diffraction patterns of the (A) Ti-0.40O and (B) Ti-0.40O-1.60Cu alloys at the as-fabricated, ~6% strain, and fractured states.


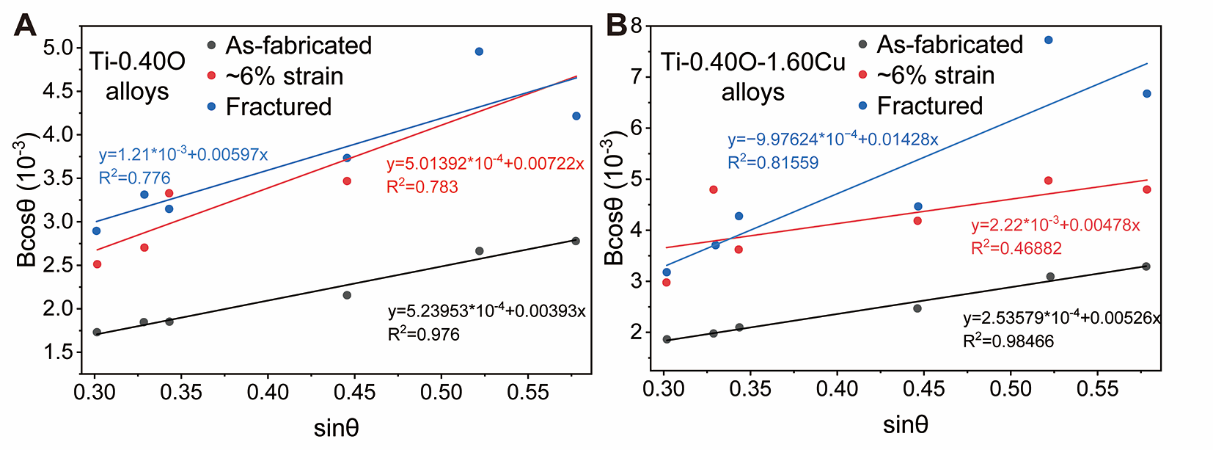


**Figure. S18.** The linear fitting based on Williamson-Hall equation for estimating the dislocation density, where the parameters are collected from Fig. S17. (A) Ti-0.40O alloy. (B) Ti-0.40O-1.60Cu alloy.


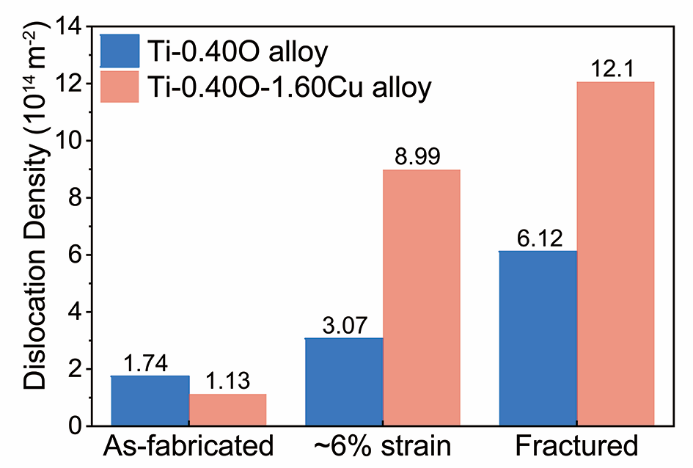


**Figure. S19.** The estimated dislocation densities of the Ti-0.40O and Ti-0.40O-1.60Cu alloys at different deformation states.
