# Supplementary figures and images for: Addressing Oxygen Embrittlement in Additively Manufactured Titanium via Cu‐Mediated Interstitial Site Engineering
Source: Adv Sci (Weinh). 2026 Jan 29;13(19):e19184. doi: 10.1002/advs.202519184 (PMC13045232; doi:10.1002/advs.202519184)

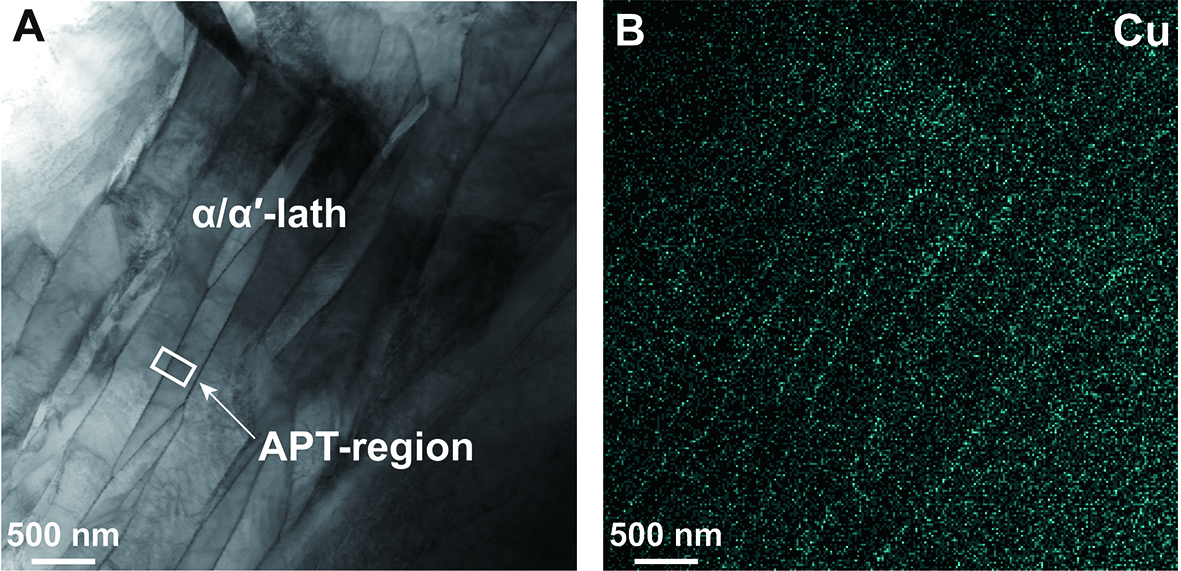

Supplement: Supplementary file 2 — Supporting File 2: advs74042‐sup‐0002‐FigureS1‐S19.zip. [file ADVS-13-e19184-s001.zip › Fig. S1.tif]

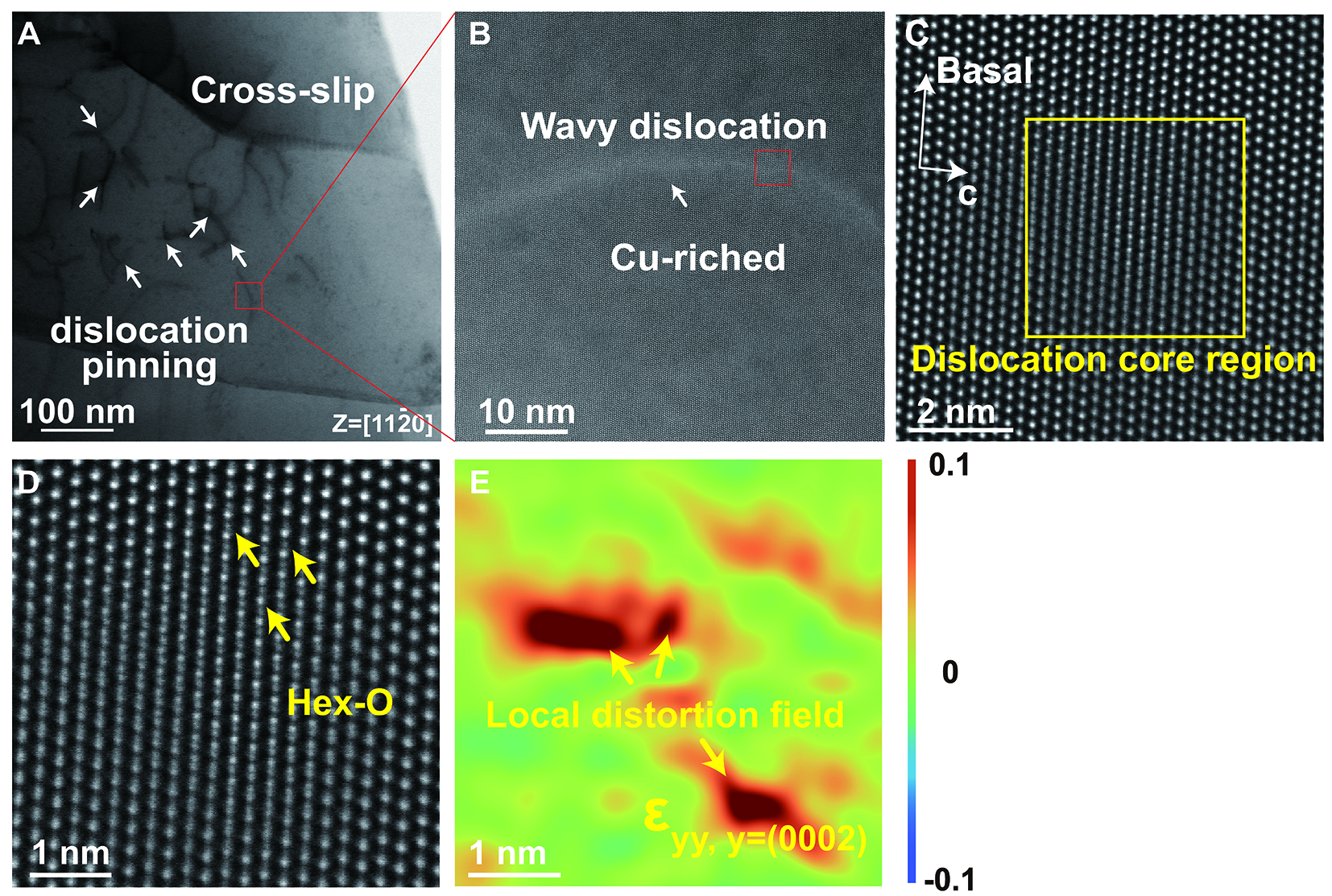

Supplement: Supplementary file 2 — Supporting File 2: advs74042‐sup‐0002‐FigureS1‐S19.zip. [file ADVS-13-e19184-s001.zip › Fig. S10.tif]

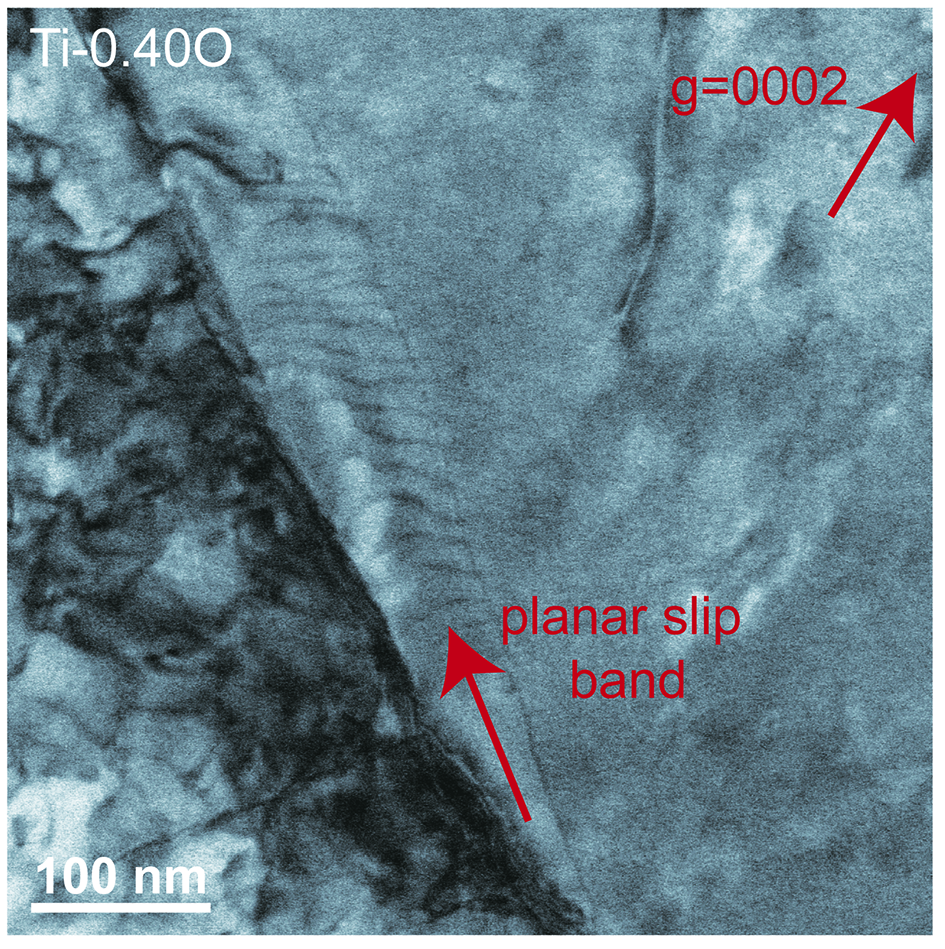

Supplement: Supplementary file 2 — Supporting File 2: advs74042‐sup‐0002‐FigureS1‐S19.zip. [file ADVS-13-e19184-s001.zip › Fig. S11.tif]

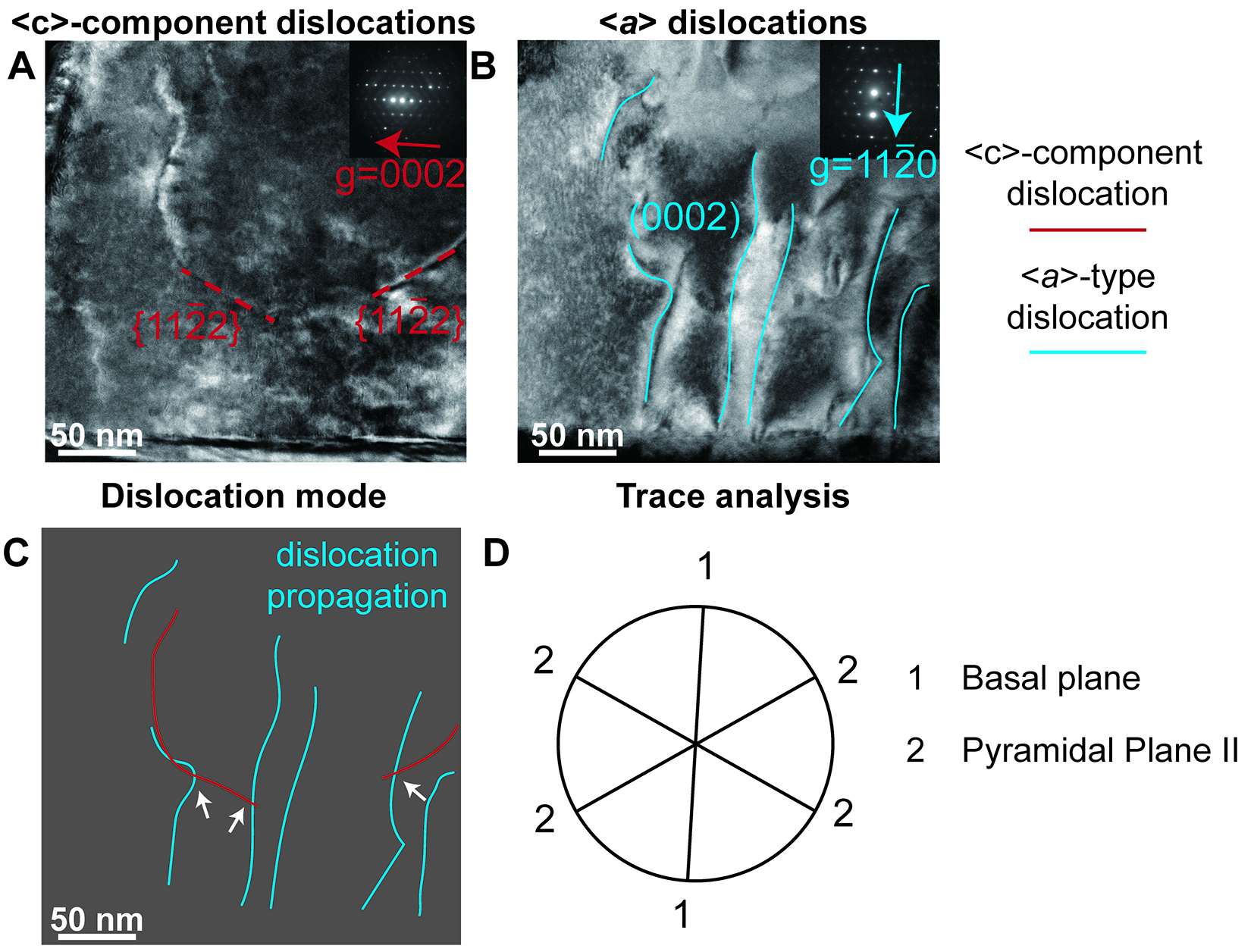

Supplement: Supplementary file 2 — Supporting File 2: advs74042‐sup‐0002‐FigureS1‐S19.zip. [file ADVS-13-e19184-s001.zip › Fig. S12.tif]

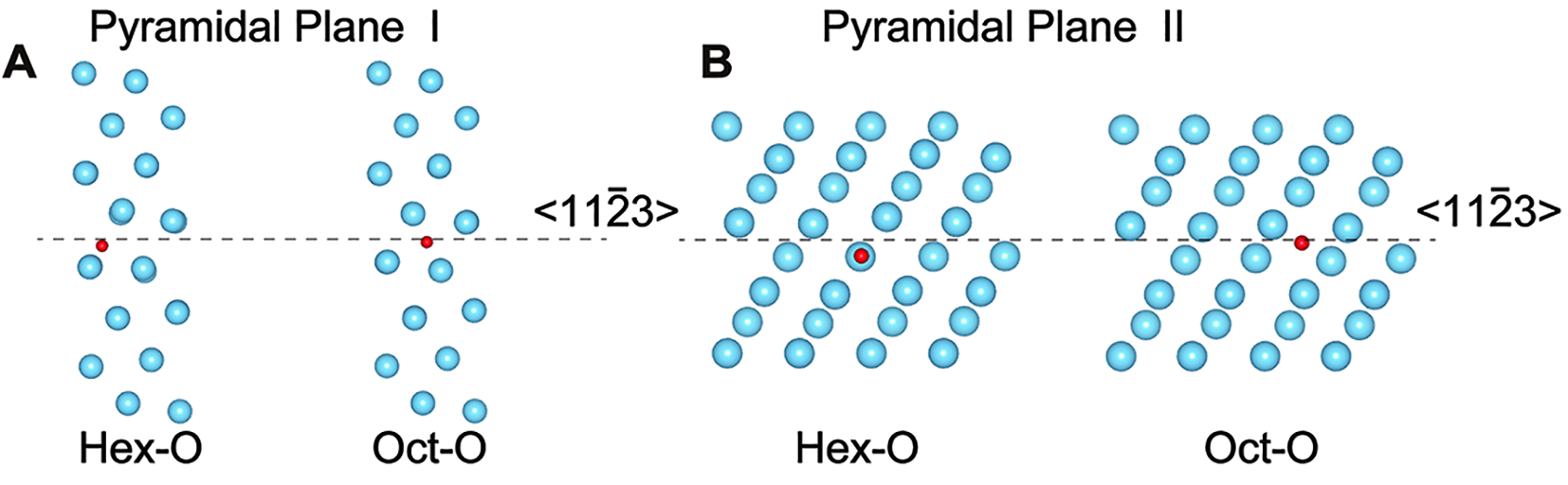

Supplement: Supplementary file 2 — Supporting File 2: advs74042‐sup‐0002‐FigureS1‐S19.zip. [file ADVS-13-e19184-s001.zip › Fig. S13.tif]

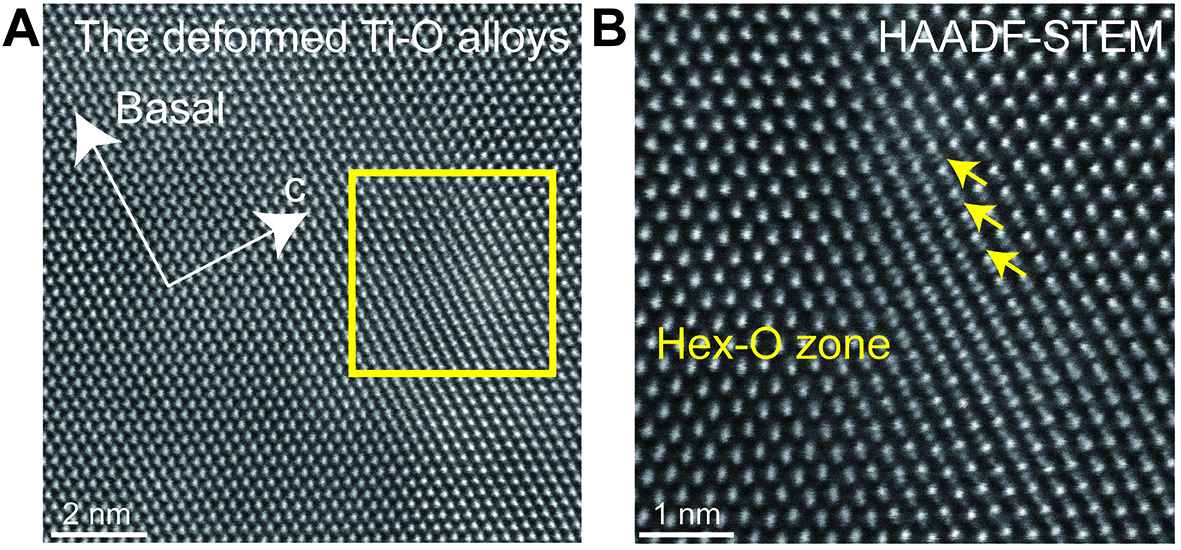

Supplement: Supplementary file 2 — Supporting File 2: advs74042‐sup‐0002‐FigureS1‐S19.zip. [file ADVS-13-e19184-s001.zip › Fig. S14.tif]

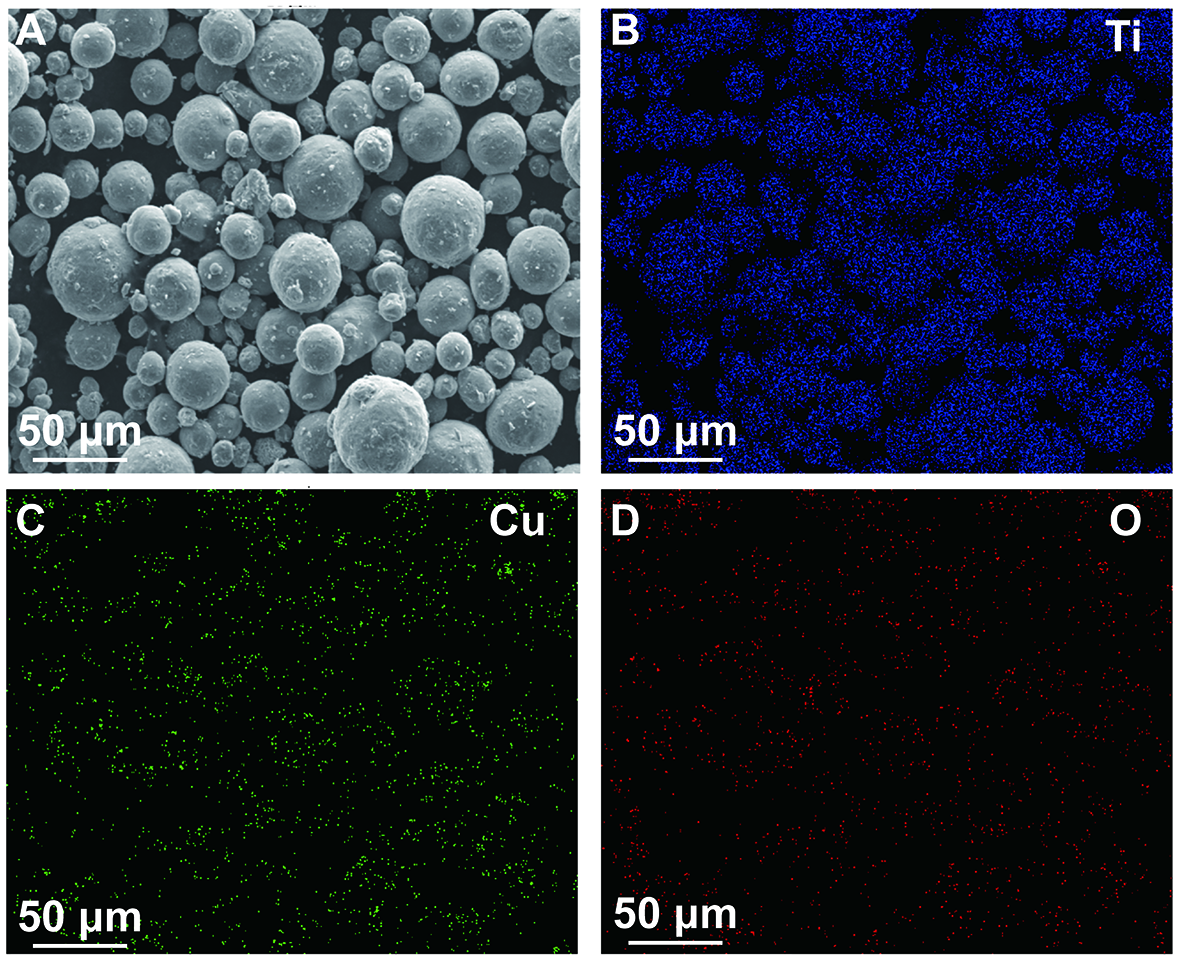

Supplement: Supplementary file 2 — Supporting File 2: advs74042‐sup‐0002‐FigureS1‐S19.zip. [file ADVS-13-e19184-s001.zip › Fig. S15.tif]

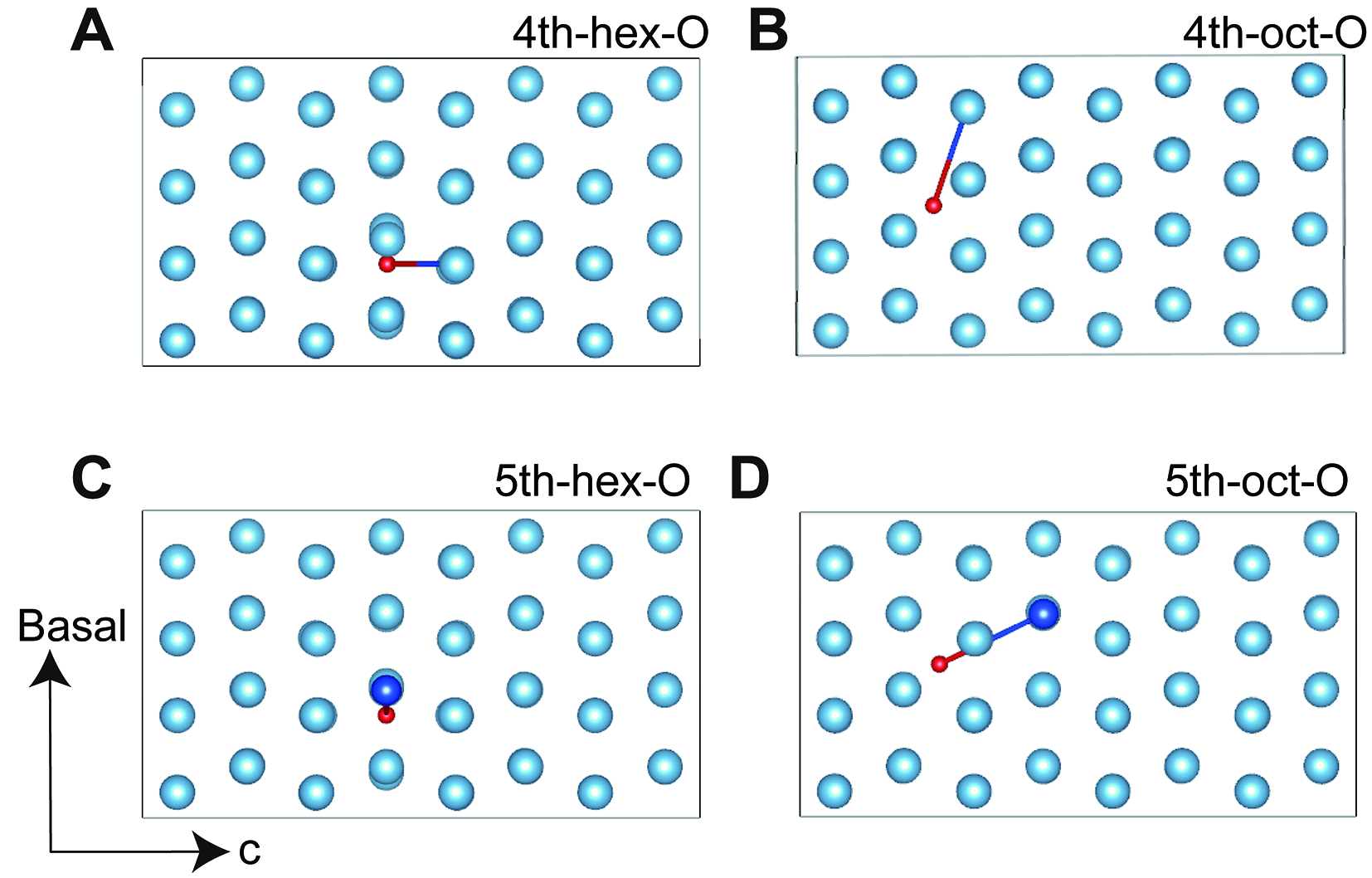

Supplement: Supplementary file 2 — Supporting File 2: advs74042‐sup‐0002‐FigureS1‐S19.zip. [file ADVS-13-e19184-s001.zip › Fig. S16.tif]

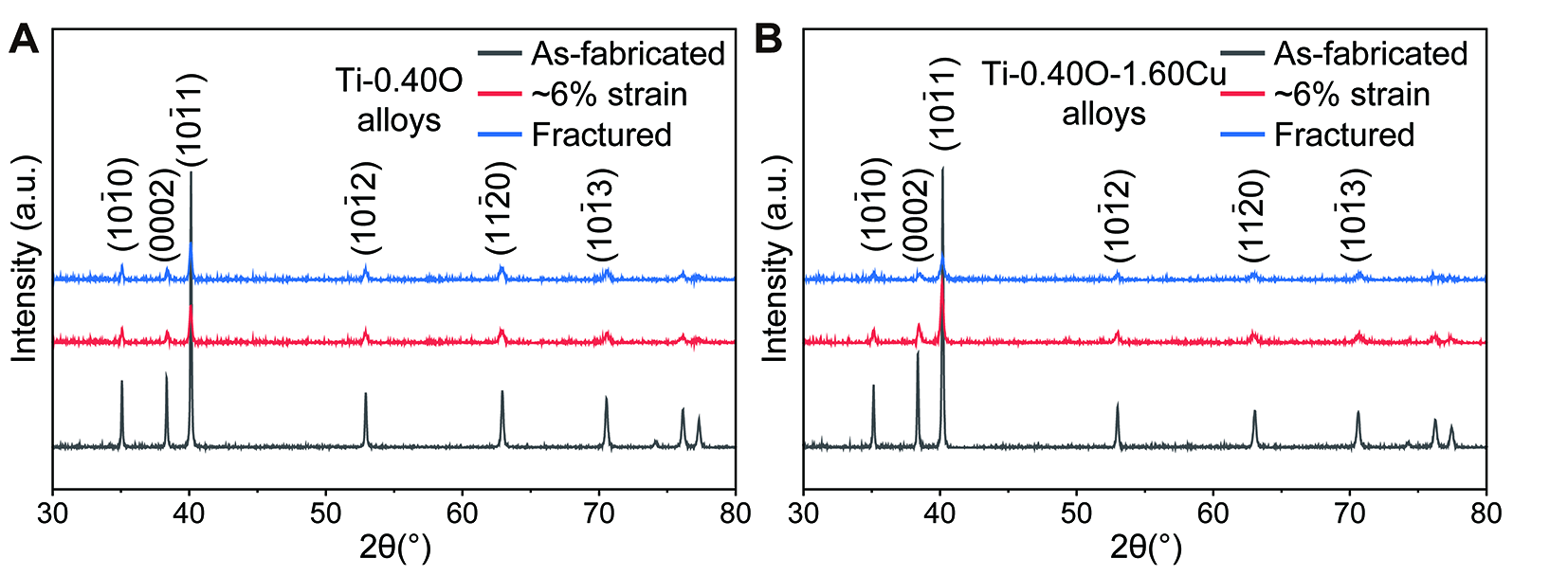

Supplement: Supplementary file 2 — Supporting File 2: advs74042‐sup‐0002‐FigureS1‐S19.zip. [file ADVS-13-e19184-s001.zip › Fig. S17.tif]

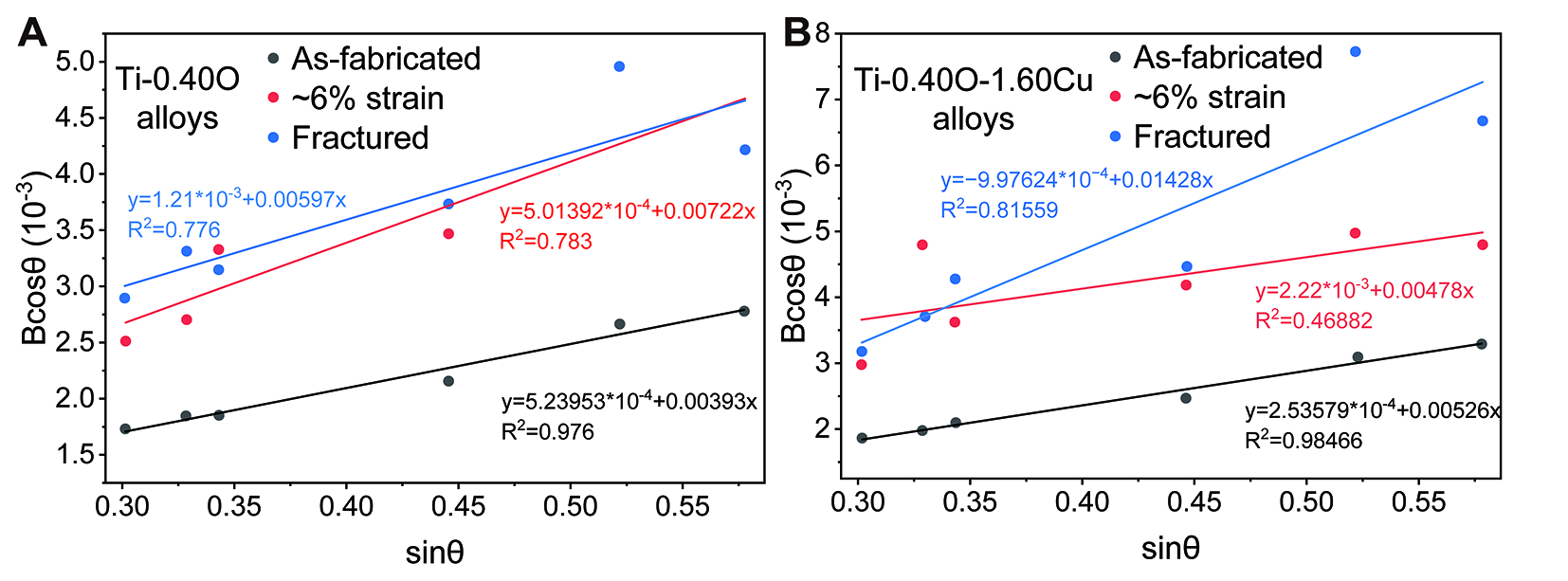

Supplement: Supplementary file 2 — Supporting File 2: advs74042‐sup‐0002‐FigureS1‐S19.zip. [file ADVS-13-e19184-s001.zip › Fig. S18.tif]

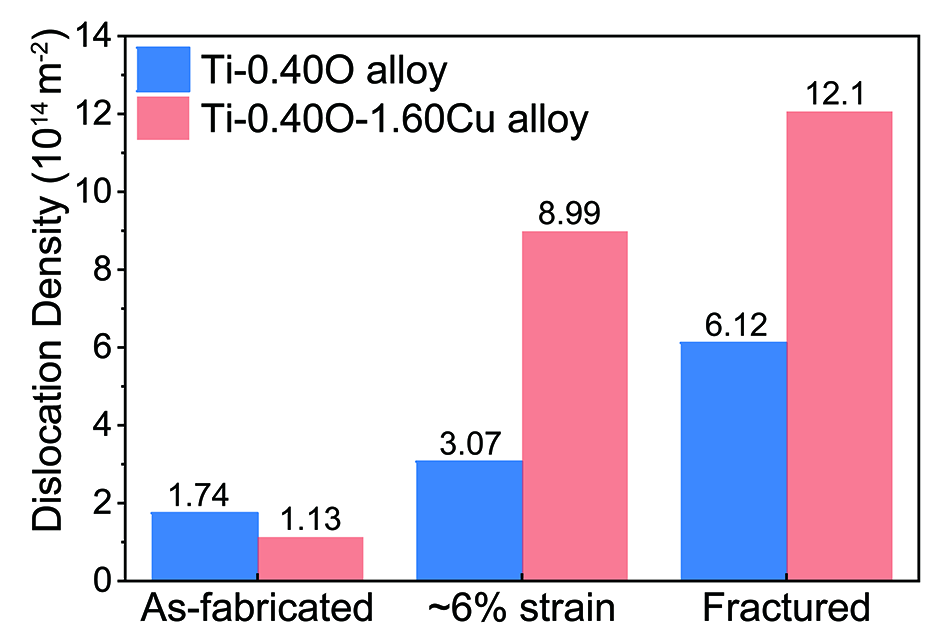

Supplement: Supplementary file 2 — Supporting File 2: advs74042‐sup‐0002‐FigureS1‐S19.zip. [file ADVS-13-e19184-s001.zip › Fig. S19.tif]

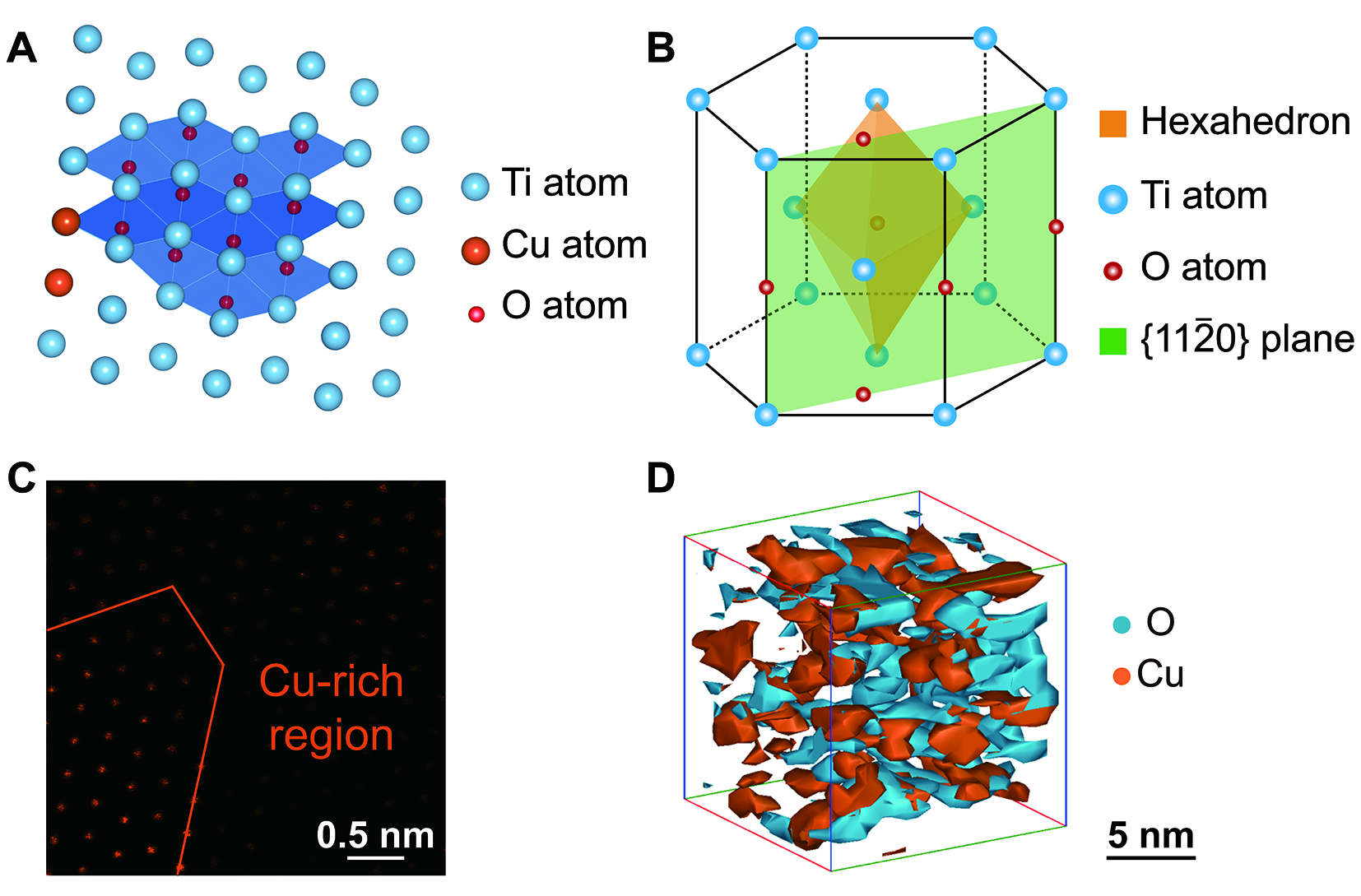

Supplement: Supplementary file 2 — Supporting File 2: advs74042‐sup‐0002‐FigureS1‐S19.zip. [file ADVS-13-e19184-s001.zip › Fig. S2.tif]

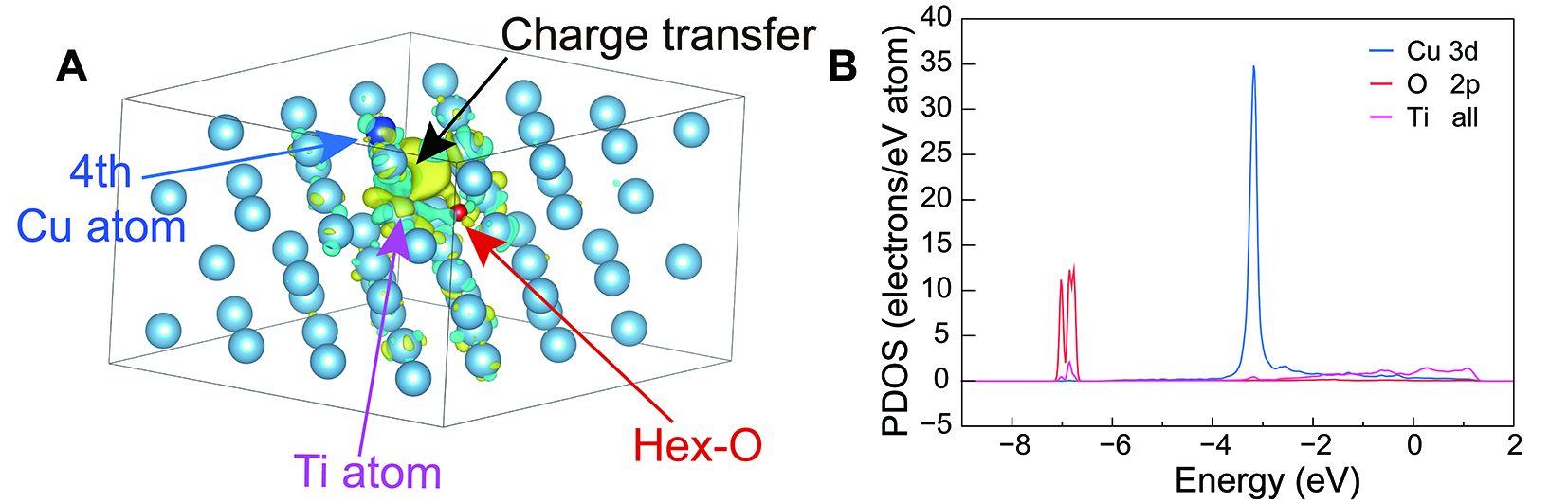

Supplement: Supplementary file 2 — Supporting File 2: advs74042‐sup‐0002‐FigureS1‐S19.zip. [file ADVS-13-e19184-s001.zip › Fig. S3.tif]

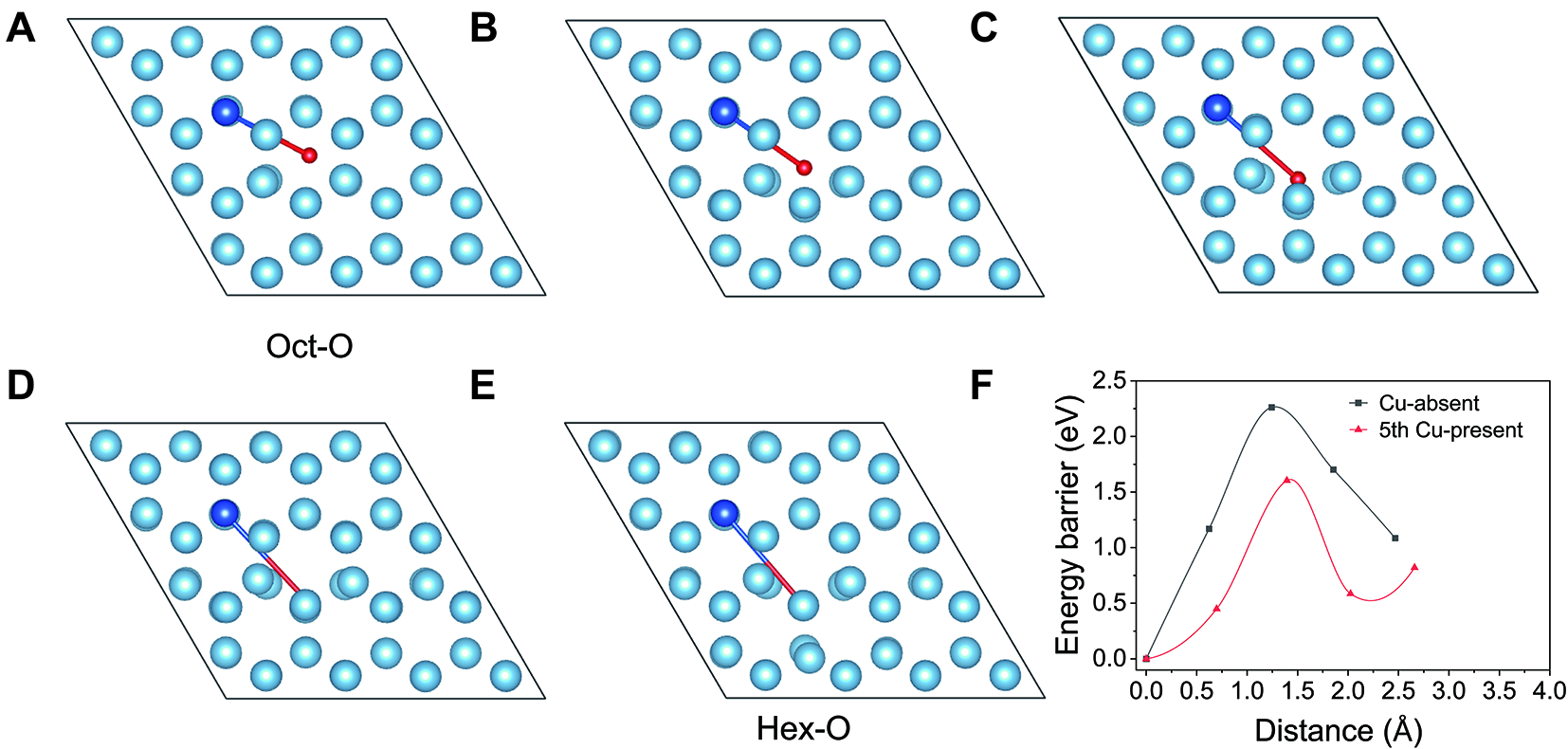

Supplement: Supplementary file 2 — Supporting File 2: advs74042‐sup‐0002‐FigureS1‐S19.zip. [file ADVS-13-e19184-s001.zip › Fig. S4.tif]

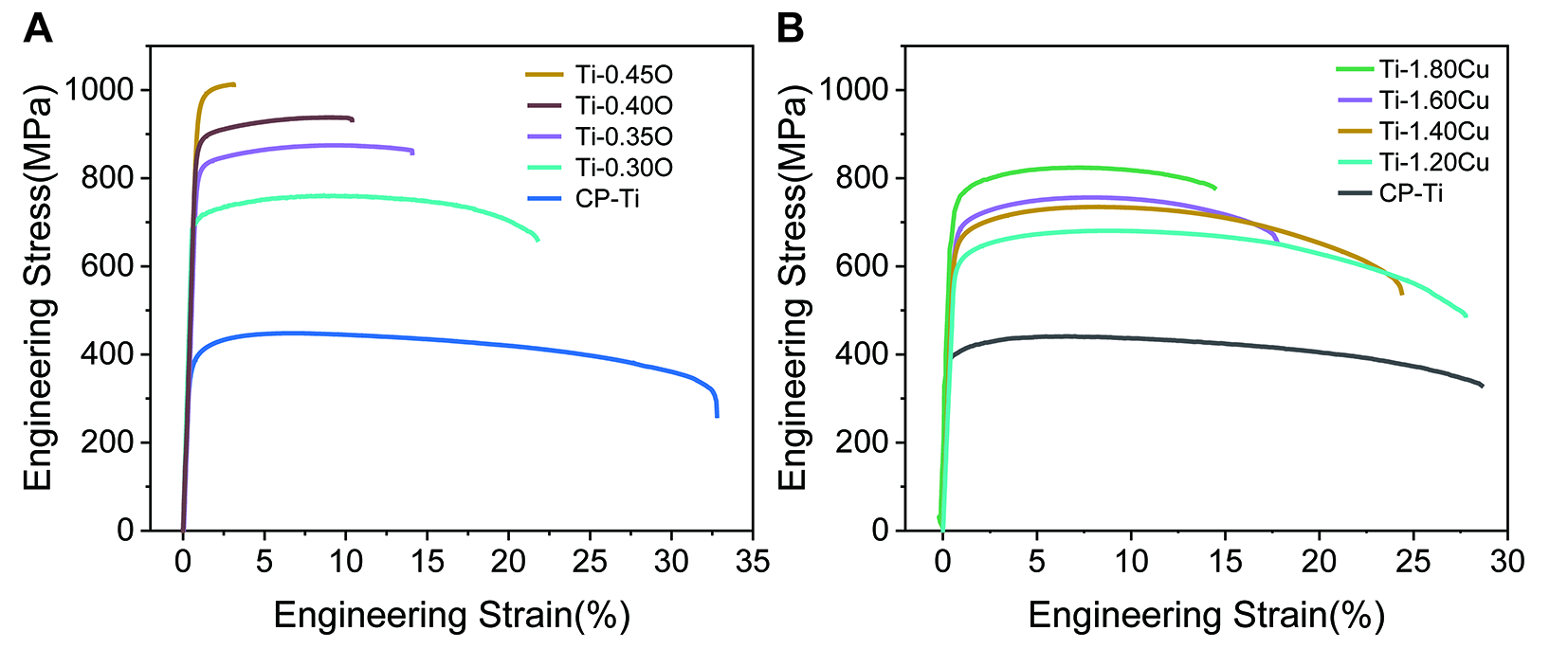

Supplement: Supplementary file 2 — Supporting File 2: advs74042‐sup‐0002‐FigureS1‐S19.zip. [file ADVS-13-e19184-s001.zip › Fig. S5.tif]

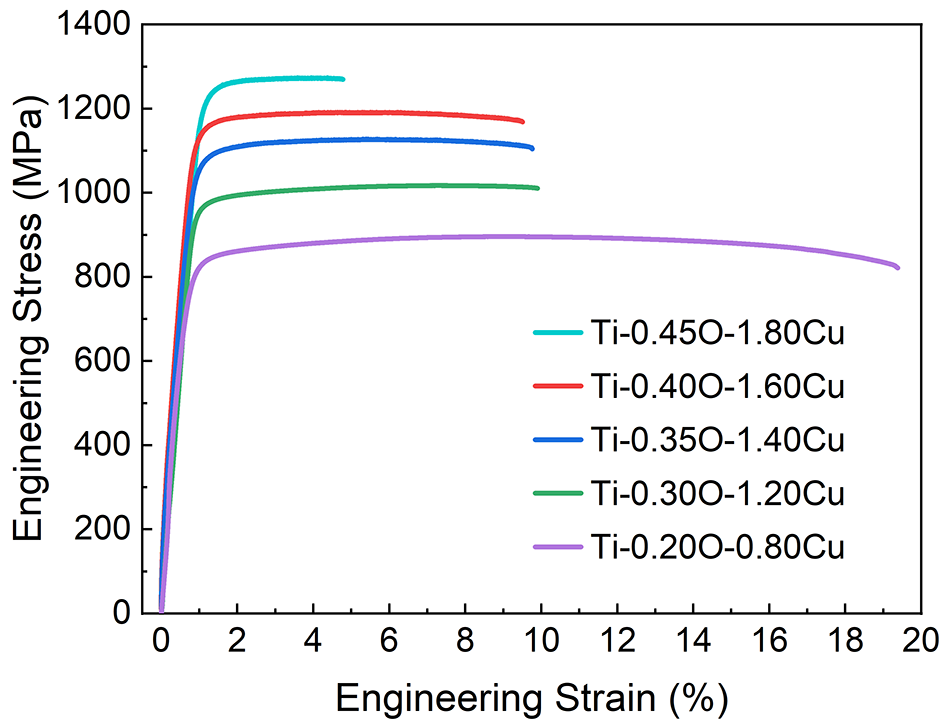

Supplement: Supplementary file 2 — Supporting File 2: advs74042‐sup‐0002‐FigureS1‐S19.zip. [file ADVS-13-e19184-s001.zip › Fig. S6.tif]

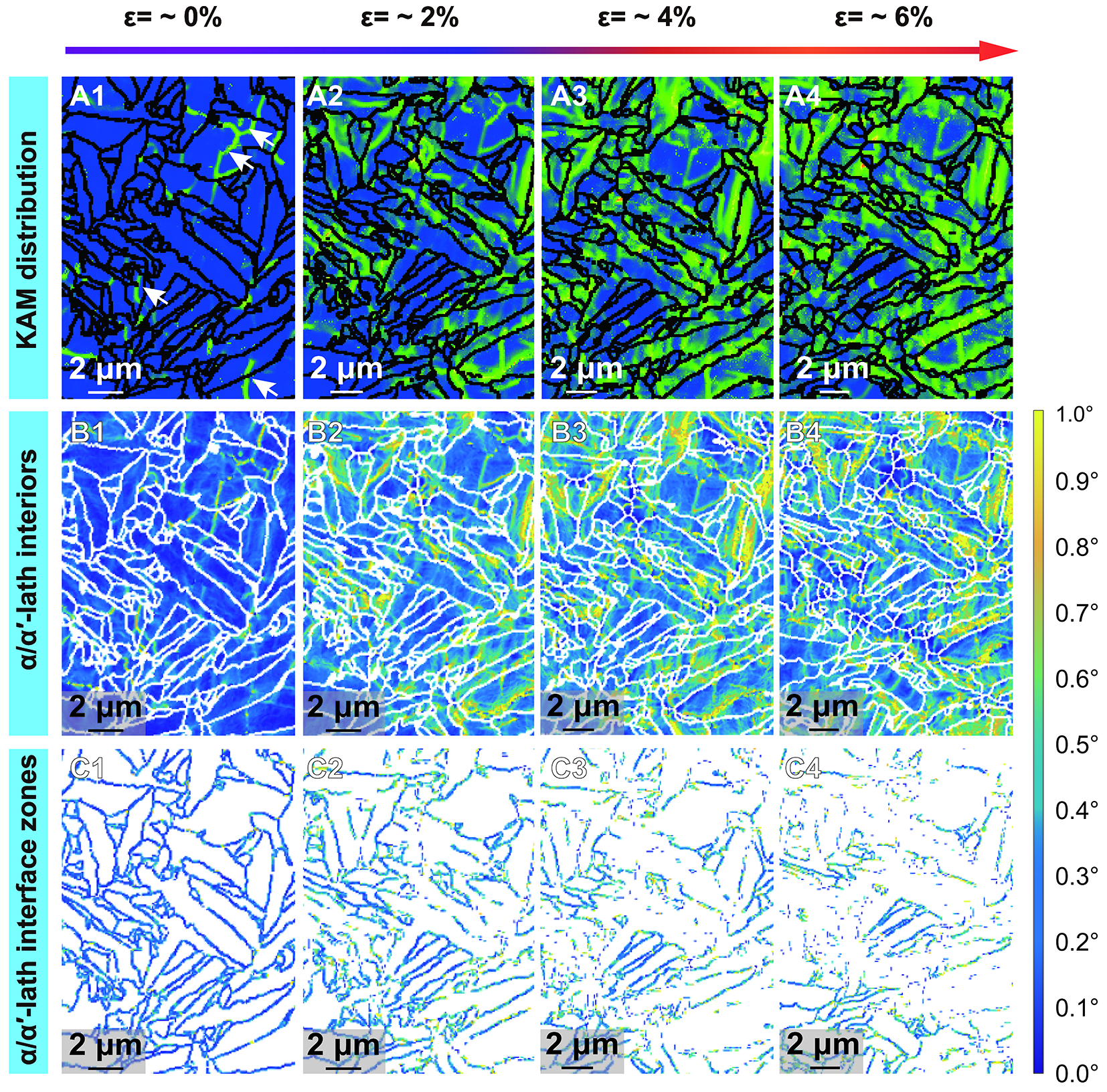

Supplement: Supplementary file 2 — Supporting File 2: advs74042‐sup‐0002‐FigureS1‐S19.zip. [file ADVS-13-e19184-s001.zip › Fig. S7.tif]

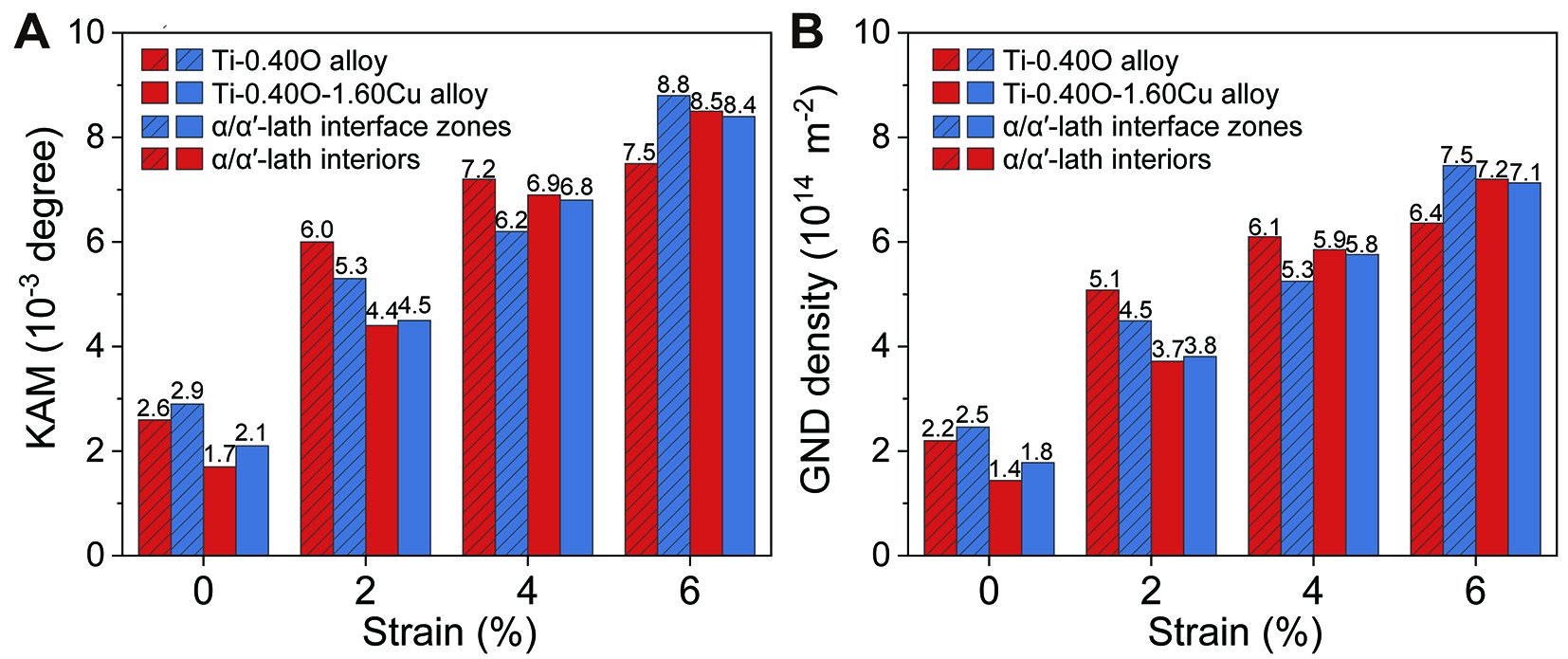

Supplement: Supplementary file 2 — Supporting File 2: advs74042‐sup‐0002‐FigureS1‐S19.zip. [file ADVS-13-e19184-s001.zip › Fig. S8.tif]

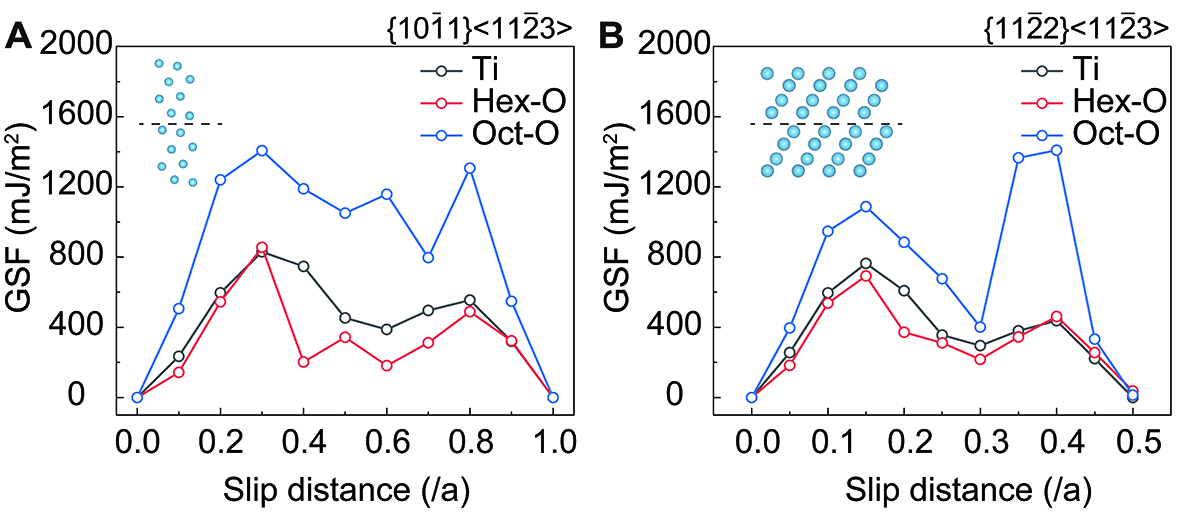

Supplement: Supplementary file 2 — Supporting File 2: advs74042‐sup‐0002‐FigureS1‐S19.zip. [file ADVS-13-e19184-s001.zip › Fig. S9.tif]
